# Design and Synthesis of Bis-Chalcones as Curcumin Simplified Analogs and Assessment of Their Antiproliferative Activities Against Human Lung Cancer Cells and *Trypanosoma cruzi* Amastigotes †

**DOI:** 10.3390/ph18040456

**Published:** 2025-03-24

**Authors:** Gabriela Alves de Souza, Lorrane de Souza Chaves, Afonso Santine M. M. Velez, Jorge Lucas F. Lacerda, Paulo Pitasse-Santos, Jayane Clys Conceição dos Santos, Otávio Augusto Chaves, Carlos Serpa, Raphael do Carmo Valente, Leonardo Marques da Fonseca, Marcos André Rodrigues da Costa Santos, Jhenifer Santos dos Reis, Carlos Antônio do Nascimento Santos, Lucia Mendonça-Previato, Jose Osvaldo Previato, Celio Geraldo Freire-de-Lima, Debora Decoté-Ricardo, Leonardo Freire-de-Lima, Marco Edilson Freire de Lima

**Affiliations:** 1 Departamento de Química Orgânica, Instituto de Química, Universidade Federal Rural do Rio de Janeiro, Seropédica 23.897-000, RJ, Brazil; gabrielakrt@hotmail.com (G.A.d.S.); afonsosv30@gmail.com (A.S.M.M.V.); jl.lacerda4@gmail.com (J.L.F.L.); jaycly@ufrrj.br (J.C.C.d.S.); 2 Programa de Pós-Graduação em Ciências Biológicas (Biofísica), Instituto de Biofísica Carlos Chagas Filho, Universidade Federal do Rio de Janeiro, Rio de Janeiro 21941-902, RJ, Brazil; lochaves8@gmail.com (L.d.S.C.); lfonseca@biof.ufrj.br (L.M.d.F.); rodrigues8mr@gmail.com (M.A.R.d.C.S.); jhnffrrs8@gmail.com (J.S.d.R.); cansantos.bio@gmail.com (C.A.d.N.S.); luciamp@biof.ufrj.br (L.M.-P.); previato@biof.ufrj.br (J.O.P.); celio@biof.ufrj.br (C.G.F.-d.-L.); 3 Programa de Pós-Graduação em Farmacologia e Química Medicinal, Instituto de Ciências Biomédicas, Universidade Federal do Rio de Janeiro, Rio de Janeiro 21941-902, RJ, Brazil; 4 Leicester Institute of Structural and Chemical Biology, University of Leicester, Leicester LE1 7HB, UK; paulopitasse@gmail.com; 5 School of Chemistry, University of Leicester, Leicester LE1 7RH, UK; 6 Department of Chemistry, Coimbra Chemistry Centre-Institute of Molecular Science, University of Coimbra, 3004-535 Coimbra, Portugal; otavioaugustochaves@gmail.com (O.A.C.); serpasoa@ci.uc.pt (C.S.); 7 Laboratory of Immunopharmacology, Centro de Pesquisa, Inovação e Vigilância em COVID-19 e Emergências Sanitárias, Oswaldo Cruz Institute, Rio de Janeiro 21040-361, RJ, Brazil; 8 Campus Duque de Caxias Professor Geraldo Cidade, Universidade Federal do Rio de Janeiro, Duque de Caxias 25.240-005, RJ, Brazil; raphael.valente@caxias.ufrj.br; 9 Curso de Medicina, Universidade Castelo Branco, Rio de Janeiro 21.710-255, RJ, Brazil; 10 Departamento de Microbiologia e Imunologia Veterinária, Instituto de Veterinária, Universidade Federal Rural do Rio de Janeiro, Seropédica 23.897-000, RJ, Brazil; decotericardo@ufrrj.br; 11 Programa de Pós-Graduação em Ciências Morfológicas, Instituto de Ciências Biomédicas, Universidade Federal do Rio de Janeiro, Rio de Janeiro 21941-902, RJ, Brazil

**Keywords:** molecular simplification, antitumor drugs, antiparasitic drugs, chemotherapy, Chagas disease

## Abstract

**Background:** Anticancer therapies represent the primary treatment option for a significant number of cancer patients globally; however, many of these treatments are associated with severe side effects as they target molecular structures present in both cancerous and healthy cells. In a similar context, the treatment of Chagas disease, a neglected tropical illness, is hindered by the high toxicity of the currently available drugs. Researchers are increasingly focusing on the development of safer and more selective alternatives, with natural compounds being studied as potential starting points for the creation of more effective drug candidates with a favorable therapeutic index. **Objectives:** The aim of this study was to design simplified curcumin-derived structures that preserved or enhanced their therapeutic activity against human lung cancer cell lines and *T. cruzi*, while also improving bioavailability and minimizing toxicity. **Methods:** In this study, curcumin and two natural curcuminoids inspired the synthesis of a chalcone and a set of bis-chalcones, compound classes known for their enhanced stability compared with their natural parent derivatives. The synthetic strategy used was the acid-catalyzed aldol condensation reaction. The stability profiles, IC_50_ values against A549 and H460 tumor cell lines, and trypanocidal activity against *T. cruzi* amastigotes of these derivatives were assessed. **Results:** The synthesized derivatives exhibited improved stability compared with the parent compounds, along with lower IC_50_ values in both A549 and H460 tumor cell lines. Additionally, one of the new analogs showed promising trypanocidal activity against *T. cruzi* amastigotes. **Conclusions:** This study provides a potential pathway toward the development of more effective and less toxic treatments for both cancer and Chagas disease. The simplified curcumin derivatives represent a promising foundation for designing new therapeutic agents with improved bioavailability and efficacy.

## 1. Introduction

The growth processes of protozoan parasites and cancer cells in the respective host organisms share several similarities. The main one is the accelerated process of cell division observed in both. Additionally, the closeness between the immune system evasion strategies used by parasites and tumors to develop and even spread to other tissues and organs is well described in the literature [1]. Biochemical processes present in parasites of the trypanosomatid family, such as *Trypanosoma cruzi*, *Trypanosoma brucei*, and *Leishmania sp*., resemble those present in tumor lineages, so it is possible to trace a correlation between the antitumor and antitrypanosomatid activities of several drugs. Taxol [2,3], doxorubicin [4], tamoxifen [5], cytarabine, and 5-fluorouracil [6] are among the examples of drugs active against both cancer and trypanosomatid cells. In addition, several studies have been described in recent years proposing the evaluation of new chemical entities against both tumor cells and trypanosomatids, including the study of mechanisms of action in homologous biochemical targets present in the different cell types evaluated [6,7,8,9].

Cancer is the second leading cause of death globally. It is responsible for killing millions of people worldwide. Among its various forms, lung cancer stands out as one of the most pervasive and lethal [10]. This disease does not discriminate, affecting people regardless of lifestyle, gender, and age. Lung cancer develops when abnormal cells in the lungs grow uncontrollably, forming tumors that can interfere with the organ’s vital functions [11]. It is often asymptomatic in the early stages, progressing until it reaches advanced levels. By then, cancer has frequently spread beyond the lungs, reducing the effectiveness of treatment options and diminishing survival rates [12].

The fight against lung cancer includes a range of treatments, each with its own set of benefits and harms. Surgery aims to remove the tumor and/or affected tissue, but it is only viable for localized cases and carries risks of complications [13]. Chemotherapy, which is a systemic method of treatment, involves the use of potent drugs to destroy transformed cells throughout the body. However, most of the time, it can also cause damage to healthy cells, leading to debilitating side effects [14]. Radiation therapy employs high-energy beams to target and shrink tumors, yet it too can cause adverse reactions, such as skin irritation and difficulty swallowing [15]. 

Despite advancements in medical science, the quest for more effective and less toxic treatments for lung cancer persists. Researchers are exploring innovative approaches, including identifying and developing selective and potent natural products [16]. These compounds, derived from plants, marine organisms, and other sources, possess diverse biological activities that hold promise in combating cancer cells while sparing healthy tissues [17].

Among the products of natural origin that have been investigated over the last fifteen years and have attracted the attention of researchers is curcumin, the active compound found in turmeric (powdered dry rhizomes of *Curcuma longa*). Its remarkable properties extend beyond its culinary use as a spice; curcumin exhibits many biological activities, including antioxidant, anti-inflammatory, and anticancer effects [18,19,20]. Studies have shown that curcumin can inhibit the proliferation of cancer cells, induce apoptosis, suppress angiogenesis, and inhibit signaling pathways associated with the epithelial-to-mesenchymal transition process, which is closely linked to metastasis [21,22,23]. Curcumin has been found to enhance the efficacy of conventional cancer therapies, such as chemotherapy and radiation, while mitigating their associated side effects [24,25]. Additionally, curcumin and two of its natural analogs show toxic effects on both the epimastigote and amastigote forms of the hemoflagellate protozoan *Trypanosoma cruzi*, the etiologic agent of Chagas disease (CD) [26]. 

CD is endemic in areas where people live in significant economic vulnerability, mainly in South and Central America [27]. *T. cruzi* has a very complex life cycle, divided between invertebrate hosts (insect vectors) and vertebrates (some species of mammals, including humans). The protozoan life cycle presents different developmental stages in the insect vector and the mammalian host [28]. The two main developmental stages of *T. cruzi* found in the vertebrate hosts are the intracellular amastigotes and the trypomastigotes in the bloodstream. Epimastigotes are found in the vector, the hematophagous insects of the Rudiviidae family popularly known as kissing bugs [29]. The World Health Organization (WHO) data show that CD is part of a group of neglected tropical diseases (NTDs), with CD being considered one of the most neglected due to the insufficient efforts of pharmaceutical companies to develop new drugs to treat people in the chronic phase of the infection. The treatment options available for chagasic patients are limited to only two drugs: nifurtimox and benznidazole. Both options have limited therapeutic potential and low efficacy in the chronic phase of the disease and long-term treatment, in addition to presenting serious adverse effects [30,31].

**Figure 1:**
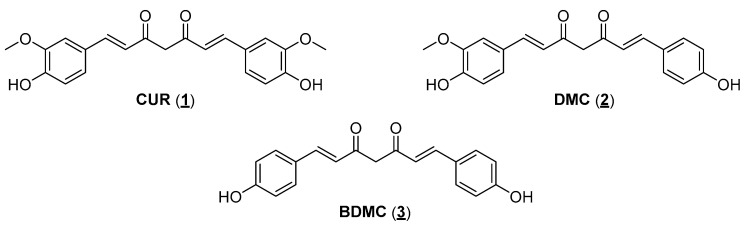
The main natural curcuminoids are from turmeric (*Curcuma longa* rhizomes).

Over the centuries, in most cultures, natural products (NPs) have been used directly as drugs to treat the various illnesses that afflict humans. NPs have continued to serve as a raw material and source of structural inspiration for designing new bioactive chemical entities [32]. A striking example of a bioactive natural product is diarylheptanoid curcumin (CUR, **1**), the main constituent of turmeric (dried rhizomes of *Curcuma longa*) [26]. Studies on the isolation of diarylheptanoids mention that the recovery of the main constituent from dry plant material is around 3%, with some examples reaching 6% CUR [33]. CUR can be obtained commercially as the main component of a mixture (approximately 75–80%) with two other curcuminoids, demethoxycurcumin (DMC, **2**) and bisdemethoxycurcumin (BDMC, 3), in percentages of 15–20% and 3–5%, respectively (Figure 1). In India, turmeric is used as a spice in many culinary preparations and in treating different diseases through the prescriptions of traditional Ayurvedic medicine. The use of turmeric has been validated over the centuries in India, and more recently, it has crossed borders as it is now a condiment used all over the world and is also one of the most consumed dietary supplements in the USA today [34]. Its use as a supplement is based on scientific studies, which associate turmeric, and mainly CUR, with a myriad of biological activities. In vitro and in vivo data demonstrate that the mixture of diarylheptanoids and their isolated components has antioxidant and anti-inflammatory activities, stimulates the immune system, and presents antitumor and even antiparasitic activities [35]. The understanding at a molecular level of the antitumor effects of CUR against different types of cancers has been improved over the last few years based on data obtained from preclinical and clinical studies [36].

**Figure 2:**
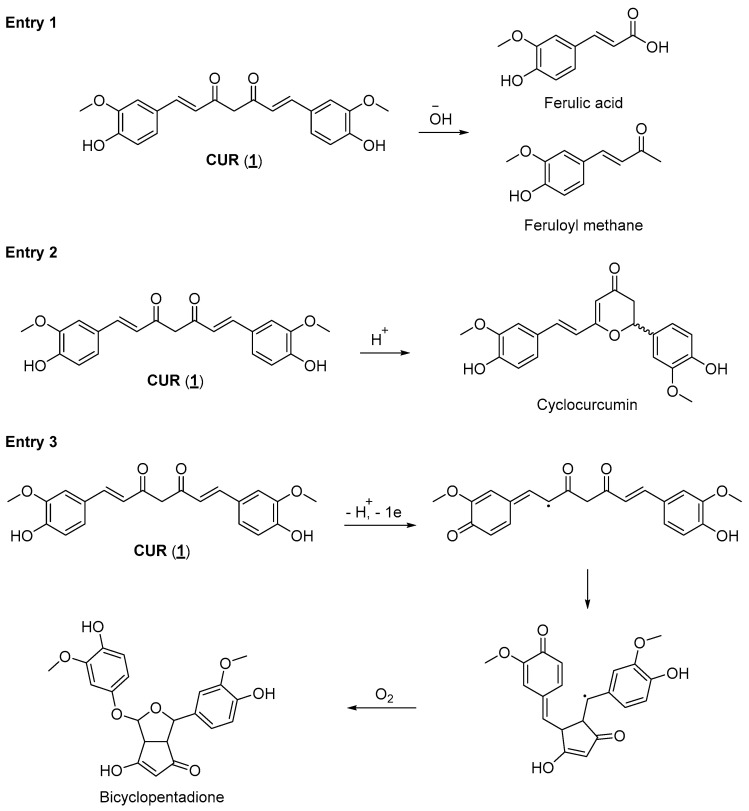
Degradation products of CUR are generated in a basic medium (entry 1), in an acidic medium (entry 2), and in its autooxidation reaction (entry 3) [41,42,43].

Regardless of the potential use of CUR as a drug with applications in several therapeutic areas, its pharmacokinetic properties limit its pharmacological utilization. Natural diarylheptanoids have low oral absorption, and the absorbed fraction is rapidly metabolized (phase I and phase II metabolism), followed by rapid elimination from the systemic circulation. Studies have shown that after oral doses of up to 12 g CUR, its plasma concentrations are deficient, which makes its therapeutic use almost impractical [37]. Attempts to obtain formulations to increase its bioavailability were, for the most part, unsuccessful in terms of obtaining a product that meets market specifications. To succeed in new formulations containing CUR, many questions still need to be answered [38,39]. Another critical issue that significantly contributes to the impossibility of using CUR as a drug, especially for oral administration, is its instability in different pH ranges. Studies described in the literature show that CUR degrades rapidly in aqueous buffer, forming various degradation products. CUR undergoes a hydrolysis reaction in alkaline aqueous media, generating products such as ferulic acid and feruloyl methane, which are less bioactive than their natural precursor (Figure 2, entry 1). When exposed to an acidic medium, CUR can cycle through an intramolecular Michael-type reaction, generating the γ-pyrone structure of cyclocurcumin (Figure 2, entry 2). Additionally, CUR reacts with molecular oxygen, forming auto-oxidation products such as bicyclopentadione (Figure 2, entry 3). Although many of these degradation products have biological activities, these differ from the activity observed in vitro for CUR [37,40]. However, curcumin’s clinical translation has been hindered by its poor bioavailability and stability, limiting its efficacy when administered orally. To overcome these challenges, researchers are exploring innovative strategies to design new compounds based on the structure of CUR, known as curcumin analogs or derivatives. These derivatives retain the beneficial properties of CUR while addressing its limitations, such as enhancing bioavailability and increasing target specificity [41,42,43].

Through structure-activity relationship studies and computational modeling, scientists identify structural modifications that can optimize curcumin analogs’ pharmacokinetic properties [44,45,46]. These modifications may include adding functional groups, altering the chemical backbone, or encapsulating in nanoparticle delivery systems, all aimed at improving the compound’s stability, solubility, and cellular uptake [46]. Moreover, researchers can rationally design analogs with enhanced potency and selectivity against specific cancer targets by elucidating the molecular mechanisms underlying curcumin’s antitumor effects. This approach holds promise for lung cancer and other malignancies where conventional therapies have fallen short [47,48]. In essence, CUR serves as a beacon of hope in pursuing innovative cancer treatments. By harnessing its intrinsic properties and leveraging modern drug design strategies, scientists aim to unlock its full therapeutic potential and usher in a new era of precision medicine in the fight against cancer [49,50].

## 2. Results and Discussion

**Figure 3:**
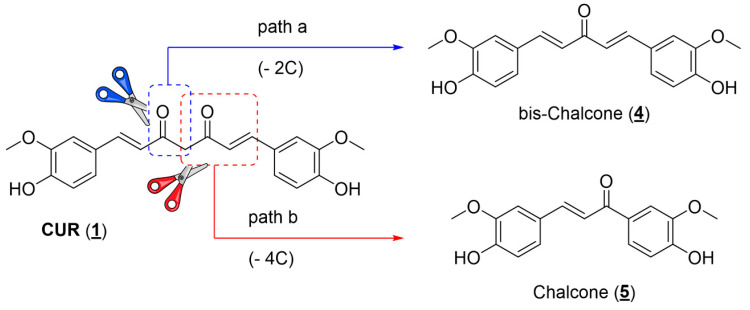
Molecular design of structurally simplified CUR analogs (**4**) and (**5**).

When developing new drugs with potential applications in cancer chemotherapy, one must consider the selectivity of action: the ratio between the drug’s activities against the cells responsible for the disease (parasites or tumor cells) and the healthy host cells. First, CUR has a low toxic potential for the human body since it is consumed in considerable amounts in food. Bayet-Robert and collaborators described the results of a phase 2 clinical trial study, in which the benefits of the combined treatment of the antitumor docetaxel and CUR were evaluated. The authors established that the recommended dose of CUR was 6000 mg/day for seven consecutive days every three weeks. In this administration regime, no unwanted effects of diarylheptanoid were observed in the tested volunteers. Thus, a relatively safe administration of this substance can be assumed [51]. Associating this information with the fact that CUR has already shown activity against a series of parasites, such as *Leishmania* sp., *T. brucei*, and *T. cruzi*, in addition to different tumor cell lines, it is worth verifying whether the other natural curcuminoids (DMC and BDMC) have comparable activities. Another relevant issue observed during the bibliographic survey is that although the literature reports several studies about the antitumor activity of CUR, most studies use the mixture of curcuminoids available in the market, composed of approximately 70% CUR, together with the other minority natural curcuminoids: DMC and BDMC (Figure 1). This mistake ultimately compromises the accuracy of the results described. In this work, the three major curcuminoid components found in the natural matrix were separated and characterized. Open-column chromatography was used to perform the separation using phosphate-buffered silica gel to avoid curcuminoid degradation during isolation [52]. The purity grade of each natural curcuminoid was determined by RP-HPLC to separately evaluate the three compounds against the two studied tumor cell lines (A549 and H460). The separation and characterization of the three main components of commercial CUR had already been previously established in work by our research group, where natural curcuminoids were adequately separated and evaluated against epimastigotes and amastigotes of *T. cruzi* (Y strain) [26]. The three natural diarylheptanoids herein assessed (**1**, **2**, and **3**) had comparable activities in the two tumor cell lines studied, presenting IC_50_ values in the same order of magnitude (Table 1). Aiming to optimize the antiproliferative activity observed for natural diarylheptanoids and obtain molecules with a possible better chemical stability profile, it was decided to employ the molecular simplification strategy in designing curcumin analogs [53]. As shown in the molecular design (Figure 3), the structure of CUR was reduced in two distinct ways: cutting out two carbon atoms, generating the structure of a bis-chalcone **4**, in which five carbon atoms were kept between the aromatic nuclei and two double bonds conjugating a single central ketone carbonyl (Figure 3, path a), and removing four carbon atoms providing chalcone **5**, which had three carbon atoms between the aromatic nuclei, preserving one of the *α,β*-unsaturated carbonyls present in CUR (Figure 3, path b). Compared with the natural prototype, the increase in chemical and metabolic stability of bis-chalcone-type curcumin analogs (diarylpentanoids) reflected their higher bioactivity. Additionally, diarylpentanoids possessed an optimized pharmacokinetic profile compared with curcuminoids [54]. Thus, these monocarbonyl derivatives have drawn attention to developing new curcumin-based agents with improved bioactivities and pharmacokinetic profiles. Several of these simplified analogs have shown anticancer and anti-inflammatory activities, and some studies have already advanced the development of new therapeutic agents and even applications in material science, such as photosensitizers and photoinitiators [55,56]. Bis-chalcones have also been evaluated for antiproliferative activity against *L. amazonensis* (promastigotes) and *T. cruzi* (epimastigotes and trypomastigotes) [57]. Likewise, the chalcone-type monocarbonyl derivatives of CUR (diarylpropanoids) have been extensively studied, showing promising activities with potential therapeutic applications in developing antitumor drugs [40,58,59,60].

**Figure 4:**
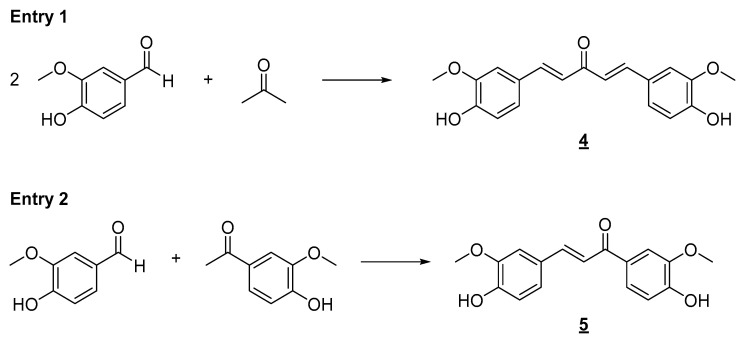
Synthesis of simplified curcumin analogs **4** and **5**. Reaction conditions: ethanol/HCl aq. (37%) 3:1, r.t., 24 h. (**4**, 61%; **5**, 53% yield).

The synthetic tool used for the preparation of the planned simplified derivatives was the Claisen–Schmidt reaction [61], which consists of cross-aldol condensation between an enolyzable aliphatic aldehyde or ketone with a non-enolyzable aromatic aldehyde. This reaction can occur in the presence of an acid or a base, generating the respective *α*,*β*-unsaturated carbonyl derivative. Due to the acidity of the phenolic hydroxyl present in the aromatic aldehyde, it was decided to carry out the condensation reaction in the presence of acid (Figure 4). The formation of a phenoxide ion on vanillin, positioned para to the aldehyde carbonyl in the aromatic ring, would negatively impact its reactivity toward the enolate. Under the conditions shown in Figure 4, it was possible to obtain bis-chalcone **4** and chalcone **5** in good yields, which were then evaluated against H460 and H549 tumor cell lines. Enones **4** and **5** have already been prepared in other previous works. Ismail and collaborators [62] assessed the antiproliferative and proapoptotic activity of bis-chalcone **4** against primary (SW480) and metastatic (SW620) human colon cancer cells [62].

The data in Table 1 demonstrates the potency optimization in simplified derivatives **4** and **5** compared with natural curcuminoids **1**–**3**. This fact is more significant in the case of diarylpentanoid **4**, which showed good antiproliferative activity against both tumor cell lines. Additionally, a remarkable improvement in the selectivity index (SI) was observed in the case of bis-chalcone 4, which did not present toxic effects against peripheral blood mononuclear cells in the higher concentration tested, elevating this simplified curcumin analog as a hit compound suitable for further structural optimizations.

**Figure 5:**
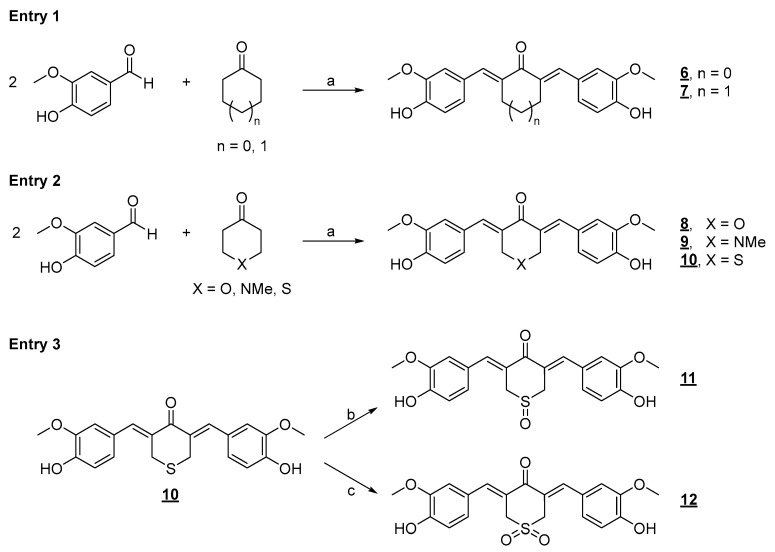
Synthetic approach to the preparation of conformationally restricted analogs. Reaction conditions: a) ethanol/HCl aq. (37%) 3:1, r.t., 24 h (23–96% yield); b) H_2_O_2_ (40 eq), acetic acid, r.t., 0.5 h (100% yield); c) OXONE^®^ (8 eq), diethylamine (5 eq), water/acetonitrile 3:1, r.t., 12 h (96% yield).

It is well established in the literature that the flexibility present in the structure of drugs causes entropic expenses in forming the drug–receptor complex. Thus, in drug design work, the conformation restriction strategy can increase the potency of a ligand (drug) by stabilizing a bioactive conformation, supporting the binding of this micromolecule to its interaction site. Another possibility is decreasing the drug degradation kinetics by blocking metabolically unstable sites or eliminating more metabolically labile conformations [63]. In derivatives **4** and **5**, a specific rigidity has already been observed due to the extension of conjugation present in the *α,β*-unsaturated system directly linked to the two aromatic nuclei. However, introducing five- and six-membered rings in the most promising analog, diarylpentanoid **4**, may improve its activity profile. First, we prepared a series of conformationally restricted analogs using this molecular optimization strategy, as shown below, including five- and six-membered hydrocarbon rings. A series of derivatives were also planned, including heteroatoms in the ketone’s six-membered ring. We used the acid-catalyzed Claisen–Schmidt synthetic approach to prepare the conformationally restricted analogs **6**–**12** (Figure 5). 

Compounds **6**–**12** were evaluated against human lung cancer cell lines (A549 and H460) and healthy human peripheral blood mononuclear cells (PBMCs) (Table 1). Cyclic diarylpentanoids **6** and **7**, as seen from the IC_50_ data in Table 1, did not show a promising profile of antiproliferative activity when compared with the acyclic derivative **4**, which was much more active in the two studied tumor cell lines. However, it was observed that cyclohexanone derivative **7** was slightly less active than cyclopentanone derivative **6**. Thus, by analyzing the IC_50_ data of these two derivatives, we concluded that the additional conformational restriction in diarylpentanoids **4** harmed the antitumor activity of the new planned derivatives against the two cell lines studied (A549 and H460). However, despite these observations, we further decided to insert rings into the diarylpentanoid framework, thereby performing the Claisen–Schmidt reaction with ketones with heteroatoms inserted in the six-membered rings. The heterocyclic ketones 4H-pyran-4-one, 1-methyl-4-piperidinone, and tetrahydrothiopyran-4-one were selected to react with vanillin by an acid-catalyzed aldol condensation reaction (Figure 5, entry 2), furnishing diarylpentanoids **8**–**10** in good yields.

**Figure 6:**
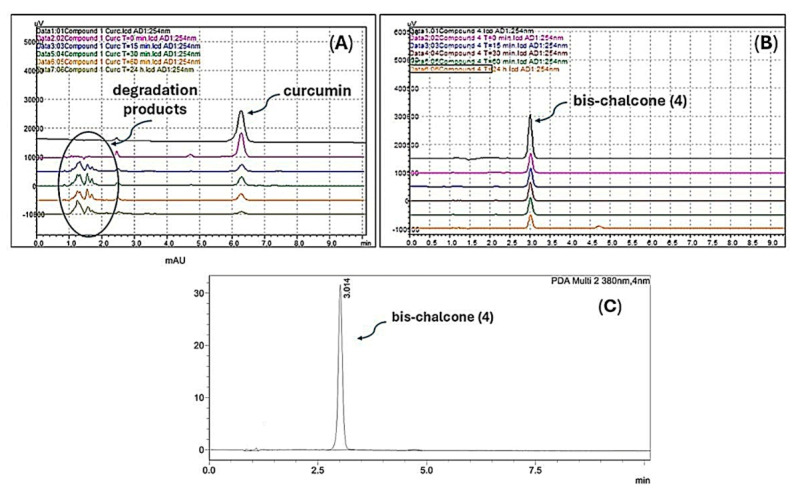
Stability of CUR **1** (**A**) and bis-chalcone **4** (**B**) in PBS (pH 7.4) at 37 °C at times of 0, 15, 30, 60 min, and 24 h, evaluated by HPLC-DAD. The chromatogram (**C**) shows the profile of bis-chalcone **4** after 24 h in PBS. All experiments were monitored at two wavelengths (254 and 380 nm).

The cyclic diarylpentanoid derivatives designed and prepared in this study were evaluated against two tumor cell lines (A549 and H460, two human lung carcinoma cell lines). The compounds were also assessed for their toxicity against human total leukocytes. The results obtained, shown in Table 1, revealed a promising activity profile for analog **8**, with IC_50_ values that indicated an almost 10- and 30-fold increase in potency compared with the parent derivative **7**. This observation suggested that an increase in electron density in this region of the molecule may be substantial for improving the antiproliferative activity exhibited by this series. For analog **9**, derived from *N*-methyl-piperidinone, although the increase in activity against the A549 cell line was not very expressive, a significant increase in H460 cells was observed with an IC_50_ of 9.89 μM, compared with the IC_50_ of 42.57 μM observed for the analog **7**. The difference in IC_50_ values for the activity against the H460 cell line was equivalent to a 4.3-fold increase in antiproliferative potency. There was also an excellent selectivity index for the evaluated molecules, which was more expressive for the H460 tumor cell line. Regarding the cyclic sulfur analog **10**, derived from the condensation of thiopyranone with vanillin (Figure 5), an increase in antitumor activity was also observed, which was more discreet than the increase observed in the activity of the two heterocyclic derivatives **8** and **9** (Table 1), which had oxygen and nitrogen in their six-membered heterocyclic rings, respectively. This modulation of the antitumor potency observed for these simplified heterocyclic analogs of CUR, diarylpentanoids **8**–**10**, may be related to the decreasing electronegativity of the heteroatoms present in the six-membered ring, causing considerable variation in the electronic density in this region of the molecule. From the IC_50_ values observed for the cyclic series **7**–**10** added to the possible interference of the structural variations implemented on the antitumor activities exhibited by these derivatives, the preparation of two derivatives through the regioselective oxidations of sulfide **10** generating sulfoxide **11** and sulfone **12** was planned (Figure 5, entry 3). The two oxidation products at the sulfur atom were suitably obtained in satisfactory yields. The antitumor activity of **11** and **12** on the two evaluated tumor cell lines validated our molecular design since there was a significant increase in antiproliferative activities against the two evaluated cell lines. The optimization was also reflected in the increase in the selectivity index of sulfoxide **11** and sulfone **12**, which were less toxic to PBMC than precursor **10** (Table 1). The toxic effect of Cisplatin (CIS), a reference chemotherapeutic agent for lung cancer treatment [64], was also analyzed on both human cancer cell lines. The results showed that CIS exhibited an IC_50_ of 6.05 μM for A549 cells and 2.96 μM for H460 cells (Table 1). The IC_50_ values for CIS on both lung cancer cell lines aligned with previously published data from other groups [65]. Although CIS is widely used for the treatment of various types of cancer, particularly lung cancer, new therapeutic strategies, including the discovery of novel molecules with potent and selective antitumor properties, need to be pursued as several studies have shown serious adverse effects caused by CIS treatment [66,67]. Chemical stability and/or improved cellular uptake of the synthesized bis-chalcones should impact their antiproliferative activity against evaluated lung cancer cell lineages compared with natural CUR **1**. Data available in the literature on the reduced stability of CUR at physiological pH encouraged us to carry out a comparative study of the stability of the natural product and bis-chalcones **4** and **8**. The results shown in Figure 6 demonstrate that in the presence of PBS (pH = 7.4) at 37 °C, approximately 70% of the CUR decomposed, generating a complex mixture of products [40,68]. The two evaluated bis-chalcones remained untouched, even after 24 h of incubation in the presence of PBS at 37 °C. Figure 6 shows the behavior of bis-chalcone **4**. Derivative **8** presented identical chemical stability under the same conditions.

**Figure 7:**
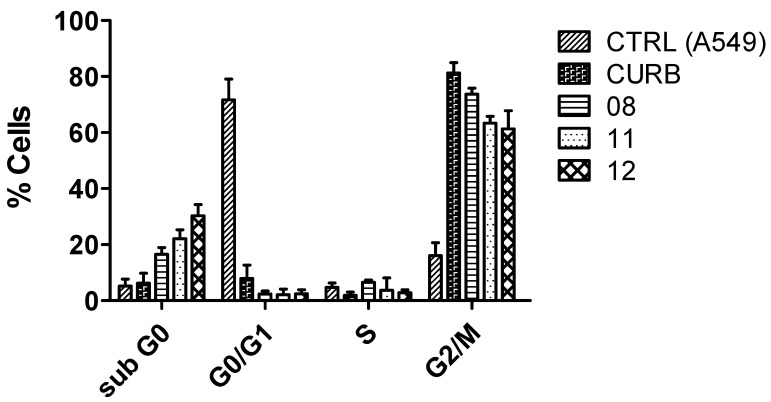
Analysis of the effect of curcuminoids with better antitumor activity on cell cycle in A549 cells. The cell cycle was measured by incorporating the DNA intercalator PI, allowing the detection of the different cell cycle phases (G0-G1, S, and G2-M). Cells were treated or not for 72 h with the IC_50_ of the more active curcuminoids on cancer cells. The distribution of cell cycle and sub-G0 phase peaks (an indicator of apoptosis) were examined by flow cytometry assay, as described in the Materials and Methods Section (Section 3)—the representative result of three individual experiments.

Investigating the interference of new molecules in the cell cycle is crucial in antitumor drug design because the cell cycle is tightly regulated and plays a central role in cellular division and growth. Cancer cells often bypass these regulatory checkpoints, leading to uncontrolled proliferation [69]. To evaluate the effects of CUR and its analogs (**8**, **11**, and **12**) with better antitumoral activity on the cell cycle of the A549 lineage, a cell cycle assay was carried out via flow cytometry using a kit containing propidium iodide (PI), Triton X100, and RNase inhibitor (Figure 7). A gate of 10,000 events was obtained by cell size and complexity, and the DNA content in each condition was detected by measuring the incorporation of the DNA intercalator PI, allowing the detection of the different cell cycle phases (G0-G1, S, and G2-M). The results depicted in Figure 7 show that in the control, nearly 75% of the cells were in the G0-G1 phase, and approximately 20% were in G2-M. With the treatments with CUR and analogs, a halt was observed in the cell cycle with a significant increase in the number of cells in the G2-M phase, ranging from 60% (molecule 12 treatment) to 80% (CUR treatment) of the cells in this phase. In addition, treatment with the analogs promoted a significant increase in the number of cells in the sub-G0 phase, reaching 30% of the total number of cells treated with bis-chalcone **12**, a phenomenon that was indicative of the induction of cell death by apoptosis [70]. 

**Figure 8:**
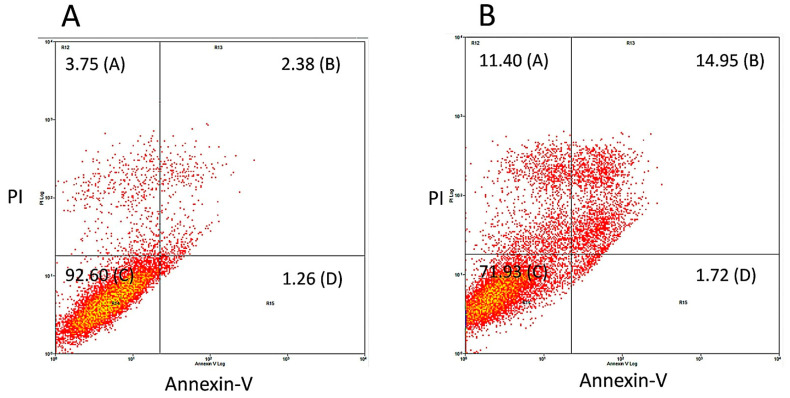
Evaluation of the cell death mechanism promoted by bis-chalcone **12**, which showed better antitumor activity in A549 cells. To evaluate cell death mechanisms, cells were treated (**B**) or not (**A**) for 48 h with **12** in its IC_50_ concentration. After treatment, the monolayers were trypsinized, and the cells were incubated with Annexin-V and PI and analyzed by flow cytometry.

To confirm that treatment with bis-chalcone **12** induced cell death by apoptosis in at least a part of the A549 cell population, a cell death assay was carried out using Annexin-V and PI dyes by flow cytometry. Annexin-V is a dye that marks phosphatidylserine residues exposed in cells undergoing apoptosis. PI, on the other hand, is a dye that penetrates cells that have lost plasma membrane integrity, a common feature of cell death by necrosis. Considering that bis-chalcone **12** was the one that demonstrated the highest effect on the induction of the sub-G0 population in the previous experiment, it was chosen to carry out the test. As depicted in Figure 8, in the incubation of 48 h, there was an increase in the percentage of double-positive cells for annexin and PI (control, A = 2.38%; treatment, B 14.98%), indicating cells in late apoptosis. This result corroborated the hypothesis that CUR analogs promote cell death in the A549 cell line and that this would preferably be by apoptosis, corroborating previous data obtained by other researchers with CUR [71,72,73].

Over the past 15 years, several papers have demonstrated that CUR can influence key cellular processes, such as the cell cycle, and the death of cancer cells by inducing apoptosis and/or necrosis. These effects contribute to the ability of curcuminoids to block tumor growth and stimulate cancer cell death, making them a promising area for cancer treatment [69,70]. The cell cycle is a tightly regulated process that controls cell division. Cell cycle dysregulation is a hallmark of cancer, leading to uncontrolled cell proliferation [71]. CUR has been shown to modulate the progression of the cell cycle in cancer cells, primarily by inducing cell cycle arrest at various stages [72]. Some studies have already demonstrated that CUR causes a G1 or G2/M phase arrest, effectively preventing the cells from progressing through the cycle and entering mitosis [73,74,75]. In addition, it has been evinced by other research groups that CUR alone or in combination with other drugs can arrest cells in the sub-G0 phase of the cell cycle, which is a stage where cells are no longer actively dividing and are considered to be in a senescence state. This arrest is often associated with induction cell death [76,77]. The effects of CUR on the cell cycle are closely associated with its ability to modulate key regulatory proteins, such as cyclins, cyclin-dependent kinases (CDKs), and checkpoint proteins like p21 and p27 [78,79,80,81]. These proteins control critical checkpoints that ensure proper cell division. Although our results demonstrate that structurally simplified curcuminoids can influence cell cycle progression, as well as the apoptosis of the lung cancer cell line A549, it is plausible to imagine that such molecules, especially **12**, which showed better activity, may modulate the expression and/or activity of cyclins and CDKs. Further studies need to be conducted to understand better the molecular pathways targeted by the curcuminoids used in the present study.

In silico predictions of pharmacokinetic properties (ADME) of small compounds are a powerful approach to suggest the potential effectiveness of a drug candidate in vivo. In this context, physicochemical, ADME parameters, and drug-likenesses of **1**–**12** were predicted using the free web server SwissADME (version 2025, http://www.swissadme.ch/index.php, accessed on 22 January 2025) [74]. As can be seen from Figure S1 in the Supplementary Materials, both passive blood–brain barrier (BBB) permeation and human intestinal absorption (HIA) were estimated by the brain or intestinal estimated permeation model (BOILED-egg) [56]. Curcuminoids **3**–**7** displayed good BBB permeability, while **1**, **2**, and **8**–**12** were expected to exhibit were expected to exhibit good intestinal absorption due to their favorable apparent polarity. Moreover, none of the compounds under study were indicated as potential substrates for the P-glycoprotein (PGP), an essential protein involved in the active efflux of substances from the central nervous system (CNS) and the gastrointestinal lumen [75].

Since the consensus lipophilicity (log P_o/w_), determined by five different predictive methods, was in the range of 2.63 to 3.05 (Table 1) and the topological polar surface area (TPSA) values were less than 140 Å^2^ (Table 1), all curcuminoids under study had potential drug-likeness, which was reinforced by the non-violations in the five drug-likeness approximations (Lipinski, Ghose, Veber, Egan, and Muegge; Table 2) [76]. Despite the curcuminoids being predicted to be not orally bioavailable due to the high quantity of unsaturations (Figure S2 in the Supplementary Materials), the molecular simplification of CUR (**1**) increased the capacity of the curcuminoids to interact as substrates to three of the five major isoforms of cytochrome P450 (CYP1A2, CYP2C9, and CYP3A4), more specifically interacting with the cofactor iron protoporphyrin IX (HEME), as suggested by molecular docking results depicted in Figure S3 in the Supplementary Materials, leading to a nontoxic effect with characteristics as lead-likeness in the medicinal chemistry [77].

To suggest the potential cancer-related targets for curcuminoids **1**–**12**, molecular docking calculations were carried out on the main proteins that were reported to interact with CUR and related compounds, i.e., epidermal growth factor receptor (EGFR, PDB code 1M17) tyrosinase kinase domain, B-cell lymphoma two protein (BCL2, PDB code 2W3L), tumor suppressor p53 (PDB code 3DCY), KIT kinase domain (KIT, PDB code 3G0E), and signal transducer and activator of transcription 3 (STAT3, PDB code 6QHD) [18]. All evaluated targets had positive docking score values (dimensionless; Table 3), suggesting that the curcuminoids under study might interact with different intracellular proteins. However, the highest docking score values were obtained for p53 (tumor suppressor gene involved in various cellular mechanisms including DNA repair, apoptosis, and cell cycle arrest) [78] and KIT (most tumors exhibit aberrant activation of this enzyme) [79], suggesting these two proteins as the main feasible targets, which were reinforced by the correlation between the docking score values with the four curcuminoids (**4**, **8**, **11**, and **12**) that had the best experimental IC_50_ values.

**Figure 9:**
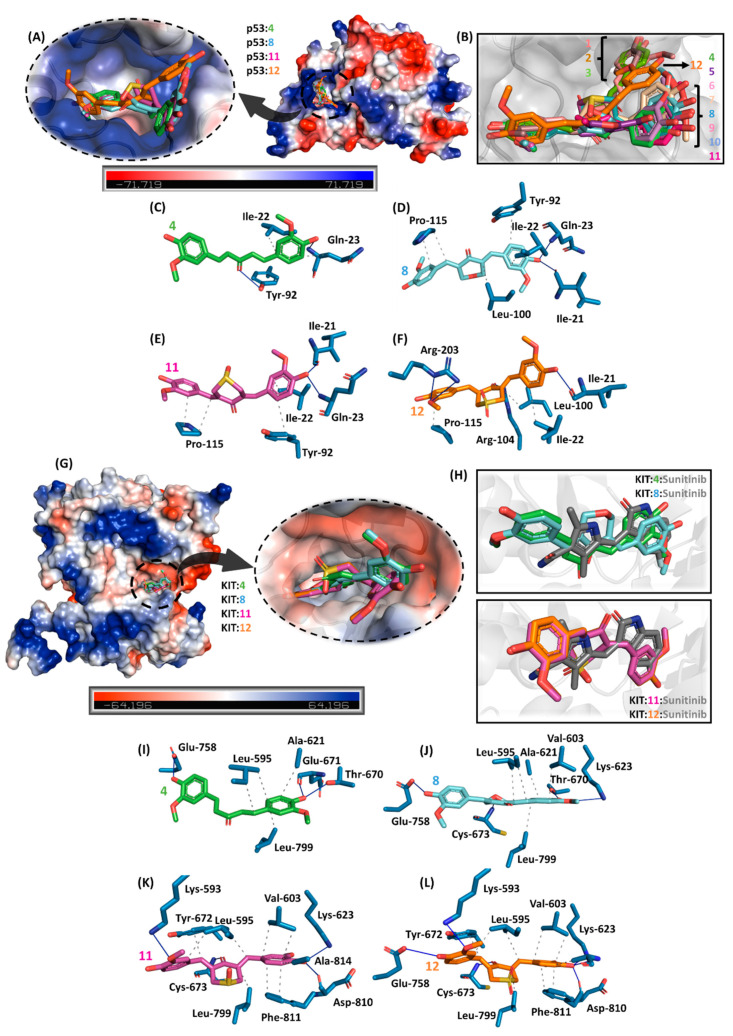
(**A**) Electrostatic potential map of p53 with the superposition of the four compounds (**4**, **8**, **11**, and **12**) with the best antitumoral profile in vitro. (**B**) Superposition of **1**–**12** into the catalytic site of p53. The main amino acid residues interact with (**C**) **4**, (**D**) **8**, (**E**) **11**, and (**F**) **12**. (**G**) Electrostatic potential map of KIT kinase domain with the superposition of **4**, **8**, **11**, and **12**. (**H**) Zoom representation of KIT:**4**:**8** and KIT:**11**:**12** in the presence of the commercial antitumoral drug sunitinib. The main amino acid residues that interact with (**I**) **4**, (**J**) **8**, (**K**) **11**, and (**L**) **12** are highlighted. Elements’ colors are as follows: oxygen, nitrogen, and sulfur are in red, dark blue, and yellow, respectively. To better visualize, hydrogen atoms were omitted. Black dots and blue lines indicate hydrophobic and hydrogen bond interactions, respectively.

Interestingly, the structural simplification of curcuminoids from **1**–**3** to **4**–**12** led the compounds to interact differently with p53 as depicted in Figure 9A,B, e.g., compound **12** which had the lowest experimental IC_50_ value to both A549 and H460 is buried in a middle way compared with **1**–**3** and **4**–**11**, interacting with the amino acid residues by hydrogen bond and hydrophobic forces (Figure 9C–F). On the other hand, curcuminoids **4**, **8**, **11**, and **12** interacted similarly compared with the commercial drug sunitinib (Figure 9G,H) by hydrogen bond and hydrophobic forces (Figure 9I–L), as well as the number of connecting points are higher to **11** and **12** than to **4** and **8**, reinforcing KIT as a feasible target.

Although tumor cells and single-celled protozoa have distinct biological origins, both share some essential biological characteristics, such as accelerated cell proliferation kinetics and similarities in critical biological mechanisms. Many anticancer or antiparasitic compounds target standard cellular processes, such as cell division, and thus, molecules that act to inhibit mitosis or DNA synthesis can interfere with the proliferation of both tumor cells and protozoa. Additionally, some antitumor agents generate reactive oxygen species (ROS), which can affect both tumor cells and protozoa, causing cellular damage to both [1]. The natural curcuminoids CUR (**1**), DMC (**2**), and BDMC (**3**) were previously tested against *T. cruzi* (Y strain). CUR had the best trypanocidal activity profile, with IC_50_ values of 10.13 μM against epimastigotes (Y strain) and inhibited amastigote release from infected mouse macrophages [26]. More recently, studies have described simplified analogs of CUR, of the diarylpentanoid type, with antiproliferative activities against *T. cruzi* (DM28c strain) and *T. b. brucei*, Lister 427 strain [80]. Din and coworkers [81,82] described nitrated diarylpentanoids’ activity against *Leishmania amazonensis* and *T. cruzi*. The nitro-diarylpentanoids described in the two works act by promoting oxidative stress in the three evolutive forms of the parasite, leading to an increase in ROS production with concomitant wear of cellular antioxidant processes. This intense oxidative impairment promotes damage to several vital structures of *T. cruzi* cells through several processes, such as lipid peroxidation and DNA fragmentation.

These antiparasitic activity results for derivatives of the diarylpentanoid class previously described in the literature added to those obtained in this work against the two cell lines of human lung tumors (H460 and A549) motivated the evaluation of the series of CUR analogs prepared in this work against intracellular amastigotes of *T. cruzi* (Tulahuen C2C4 strain containing the *lacZ* gene) [83]. The amastigotes are, clinically, the most important *T. cruzi* evolutive form as they are responsible for maintaining the infection in the invertebrate hosts and forming the so-called dormant amastigote nests in chronically infected tissues [84]. The series of conformationally restricted derivatives with six-membered heterocyclic rings **8**–**12** (Figure 5), along with CUR and the reference drug benznidazole, were evaluated against intracellular amastigotes of *T. cruzi* of the Tulahuen strain transfected with the *LacZ* gene (C2C4). This assay methodology was developed by Buckner et al. [83] and is extensively used to screen new trypanocidal candidates because it facilitates reading with the chromogenic substrate chlorophenol red-*β*-D-galactopyranoside. Analogs **8**–**12** presented IC_50_ values, as shown in Table 4, slightly more active than those of natural CUR and with trypanocidal activities comparable to that presented by the reference drug benznidazole. Due to the very flat profiles of trypanocidal activity observed for this series (Table 4), it was not possible to elaborate a more robust discussion of SAR, such as the one based on the antiproliferative action data of the same analogs against the two cancer lung cell lines (Table 1). However, regardless of the low variation in the results observed for trypanocidal activity, the results obtained on the optimization and simplification of the synthetic approach for the preparation of new entities applicable to the treatment of NTDs were of extreme importance since the accessibility and low costs of possible new drugs were essential requirements in this therapeutic area [85,86]. The results were promising, highlighting analog **9**, which presented a better activity profile, with an IC_50_ of 9.60 µM on *T. cruzi* amastigotes and a selectivity index close to 3. Thus, diarylpentanoid **9** can be seen as a hit for the development of a new family of antichagasic drugs. The comparison and calculation of selectivity indices against PBMC were not appropriate in this case due to the important differences in the time of exposure of the cells to the molecules tested, in the antiamastigote activity and cytotoxicity tests against PBMC, which were 120 h and 72 h, respectively (see the Materials and Methods Section below).

## 3. Materials and Methods

### 3.1. Chemistry

#### 3.1.1. Equipment, Reagents, and Solvents

Melting points were determined on a Fisatom 430D (São Paulo, Brazil) apparatus. The high-performance liquid chromatography (HPLC) analyses were conducted on a Shimadzu-LC20AT (Shimadzu Inc., Kyoto, Japan). Analytical conditions were as follows: column, C18, 25 m × 4.6 mm × 5 µm (Betasil-Thermo, Santa Clara, CA, USA); detector, DAD SPD-M20A, CBM-20A; oven, CTO-20A; gradient method, PDA (200–500 nm), 60% H_2_O-1%AcOH (A), 40% MeOH (B); injection volume, 20 μL; flow, 1.0 mL/min.; % transmittance. The ^1^H and ^13^C nuclear magnetic resonance (NMR) spectra were recorded on a Bruker Ultrashield Plus Spectrometer (BrukerBioSpin GmbH, Rheinstetten, Germany) operating at 500 MHz for ^1^H and 125 MHz for ^13^C. The ^1^H and ^13^C NMR shifts (δ) were reported in parts per million (ppm) concerning dimethyl sulfoxide or chloroform (DMSO-*d*_6_, 2.50 ppm for ^1^H and 39.7 ppm for ^13^C; CDCl_3_, 7.27 ppm for ^1^H and 77.00 ppm for ^13^C). Coupling constants (*J*) were reported in hertz (Hz). Signal multiplicity was assigned as singlet (s), doublet (d), doublet of doublets (dd), triplet (t), quartet (q), multiplet (m), and broad signal (brs). The liquid chromatography–high-resolution mass spectrometry (LC-HRMS) analyses were carried out on an Agilent 6530 accurate-mass quadrupole time-of-flight (Q-TOF) LC/MS system (Agilent Technologies Inc., Santa Clara, CA, USA). Analytical thin-layer chromatography (TLC) was performed on pre-coated silica gel plates (0.25 mm layer thickness) in an appropriate solvent, and the spots were visualized under UV light (254 or 356 nm).

#### 3.1.2. Separation of Natural Curcuminoids from the Commercial Curcumin

The separation and characterization of the three main natural curcuminoids present in dry *Curcuma longa* rhizomes (CUR **1**, DMC **2**, and BDMC **3**) were performed using commercial curcumin (Sigma-Aldrich, St. Lois, MO, USA). The separation methodology had already been established in previous work from our research group [26]. Successive recrystallizations properly separated the natural curcuminoids from a mixture of methanol/water (7:3), with the mother liquor enriched in DMC and BDMC being subjected to separation by column chromatography on silica-gel previously adsorbed with NaH_2_PO_4_. In this way, CUR, DMC, and BDMC were obtained in suitable amounts and pure form (see TLC analysis shown in Figure S4) for their characterization by spectroscopic analysis methods and in purities over 95% (evaluated by HPLC). The three natural diarylheptanoids, separated from commercial curcumin following the methodology previously adopted in the laboratory, presented melting points, ^1^H and ^13^C NMR spectra data, and MS data, fully compatible with those previously observed [26]. An amount of 1 g of pure CUR (**1**) (40% yield) was obtained as orange crystals with an mp of 182–185 °C (Lit., 184 °C) [26]. Demethoxycurcumin (DMC, **2**) was purified as an orange amorphous solid with a 14% yield (mass of 0.100 g). The mp was 179–181 °C (Lit., 181–182 °C) [26]. Bisdemethoxycurcumin (BDMC, **3**) was isolated as a red amorphous solid in a 3% yield (mass of 0.022 g). The mp was 232–234 °C (Lit., 232−233 °C) [26].

##### (1E,6E)-1,7-Bis(4-hydroxy-3-methoxyphenyl)hepta-1,6-dien-3,5-dione (CUR, **1**)

Data: ^1^H NMR (500 MHz, DMSO-*d_6_*) δ (ppm): 7.57–7.53 (d, 2H, *J* = 15.0 Hz); 7.32 (s, 2H); 7.15–7.16 (d, 2H, *J* = 10.0 Hz); 6.84–6.82 (d, 2H, *J* = 10.0 Hz); 6.78–6.75 (d, 2H, *J* = 15.0 Hz); 6.06 (s, 1H); 3.84 (s, 6H, -OCH_3_) (Figure S5); ^13^C NMR (DEPTQ) (125 MHz, DMSO-*d_6_*) δ (ppm): 183.63; 149.78; 148.44; 141.19; 126.77; 123.72; 121.51; 116.14; 111.68; 101.38; 56.11 (Figure S6); HRMS (ESI^−^): *m*/*z* 367.1167 [M−H]^−^ (found); *m*/*z* 367.1187 (calculated for [C_21_H_20_O_6_−H]^−^) (Figure S7).

##### (1E,6E)-1-(4-Hydroxy-3-methoxyphenyl)-7-(4-hydroxyphenyl)hepta-1,6-dien-3,5-dione (DMC, **2**)

Data were as follows: ^1^H NMR (500 MHz, DMSO-*d_6_*) δ (ppm), 10.20 (sl, 1H, -OH), 9.79 (s, 1H, -OH), 7.58–7.52 (m, 4H), 7.31 (s, 1H), 7.14–7.15 (d, 1H), 7.78–7.74 (d, 1H, *J* = 15.8Hz), 6.83–6.81 (m, 4H), 6.71–6.68 (m, 1H, *J* = 15.8Hz), 6.06 (s, 1H), and 3.68 (s, 3H, -OCH_3_) (Figure S8); ^13^C NMR (DEPTQ) (125 MHz, DMSO-*d_6_*) δ (ppm), 183.78, 183.65, 160.31, 149.85, 148.49, 141.22, 140.88, 130.86, 123.72, 121.52, 121.31, 116.41, 116.17, 111.70, 101.46, and 56.16 (Figure S9); HRMS (ESI^−^), *m*/*z* 367.1077 [M−H]^−^ (found); *m*/*z* 367.1081 (calculated for [C_20_H_18_O_5_−H]^−^) (Figure S10).

##### (1E,6E)-1,7-Bis(4-hydroxyphenyl)hepta-1,6-diene-3,5-dione (BDMC, **3**)

Data were as follows: ^1^H NMR (500 MHz, DMSO-*d_6_*) δ (ppm), 7.61–7.58 (d, *J* = 15.8 Hz, 2H), 7.52–7.51 (d, *J* = 8.8 Hz, 2H), 6.85–6.83 (d, *J* = 8.8 Hz, 2H), 6.62 (d, *J* = 15.8 Hz, 2H), and 5.97 (s, 1H) (Figure S11); ^13^C NMR (DEPTQ) (125 MHz, DMSO-*d_6_*) δ (ppm), 183.49, 159.71, 140.46, 129.74, 126.61, 120.61, 117.45, 115.49, and 100.44 (Figure S12); HRMS (ESI^−^), *m*/*z* 307.0966 [M−H]^−^ (found); *m*/*z* 307.0976 (calculated for [C_19_H_16_O_4_−H]^−^) (Figure S13).

#### 3.1.3. Chemical Synthesis

##### Synthesis of (E)-1,3-Bis(4-hydroxy-3-methoxyphenyl)prop-2-en-1-one (**5**)

Vanillin (380 mg, 2.5 mmol) and acetovanillone (415 mg, 2.5 mmol) were dissolved in a mixture of hydrochloric acid 37% (1 mL) and ethanol (3 mL). After complete consumption of vanillin, monitored by TLC, the reaction was added to a 100 mL beaker containing crushed ice. The precipitate was then vacuum-filtered and dried at room temperature.

The product was isolated as a green solid weighing 457.5 mg (1.5 mmol, 61% yield). The m.p. was 114–116 °C [87].

Data were as follows: ^1^H NMR (500 MHz, CDCl_3_/ CD_3_OD) δ 3.79–3.80 (m, 6H), 6.74 (d, 1H, *J* = 8.0 Hz), 6.78 (d, 1H, *J* = 8.0 Hz), 7.01–7.04 (m, 2H), 7.24-7.27 (m, 1H), 7.43 (s, 1H, *J* = 1.6 Hz), 7.47 (dd, 1H, *J* = 8.3, 1.6 Hz), and 7.56 (d, 2H, *J* = 15.0 Hz) (Figure S14); ^13^C NMR (DEPTQ) (125 MHz, CDCl_3_/CD_3_OD) δ 55.57, 110.50, 110.84, 114.17, 115.12, 118.66, 123.00, 123.42, 126.89, 130.30, 144.60, 147.45, 148.75, 151.05, and 189.44 (Figure S15); HRMS (ESI^+^), *m*/*z* 301.1079 [M+H]^+^ (found); *m*/*z* 301.1070 (calculated for [C_17_H_16_O_5_+H]^+^) (Figure S16).

##### General Procedure for the Synthesis of Bis-Chalcones **4** and **6**–**10**

Vanillin (760 mg, 5 mmol) and the proper ketone (2.5 mmol) were dissolved in hydrochloric acid 37% (1 mL) and ethanol (3 mL). After completely consuming vanillin, monitored by TLC, the reaction was added to a 100 mL beaker containing crushed ice. The precipitate was then vacuum-filtered and dried at room temperature.

##### (1E,4E)-1,5-Bis(4-hydroxy-3-methoxyphenyl)penta-1,4-dien-3-one (**4**)

The product was isolated as an orange solid weighing 588.8 mg (1.32 mmol, 53% yield). The mp was 97 °C (Lit., 98–99 °C) [88].

Data were as follows: ^1^H NMR (500 MHz, CDCl_3_) δ 3.98 (s, 6H), 6.96 (d, 2H, *J* = 20.0 Hz), 6.98 (d, 2H, *J* = 5.0 Hz), 7.14 (s, 2H), 7.21 (d, 2H, *J* = 10 Hz), 7.70 (d, 2H, *J* = 20.0 Hz), and 9.85 (s, 2H) (Figure S17); ^13^C NMR (DEPTQ) (125 MHz, CDCl_3_) δ 55.99, 109.76, 114.86, 123.34, 123.39, 127.47, 143.26, 146.83, 148.20, and 188.82 (Figure S18); HRMS (ESI^−^), *m*/*z* 325.1062 [M−H]^−^ (found); *m*/*z* 325.1081 (calculated for [C_19_H_18_O_5_−H]^−^)(Figure S19).

##### 2,5-Bis((E)-4-hydroxy-3-methoxybenzylidene)cyclopentan-1-one (**6**)

The product was isolated as a yellow solid weighing 631 mg (1.8 mmol, 72% yield). The mp was 190 °C (Lit., 212–214 °C) [88].

Data were as follows: NMR ¹H (500 MHz, DMSO-*d_6_*) δ (ppm), 9.67 (s, 2H), 7.37 (s, 2H), 7.25 (s, 2H), 7.17 (d, 2H, *J* = 10.0 Hz), 6.90 (d, 2H, *J* = 10.0 Hz), 3.85 (s, 6H), and 3.07 (s, 4H) (Figure S20); ^13^C NMR (DEPTQ) (125 MHz, DMSO-*d_6_*) δ, 195.24, 148.98, 148.16, 135.19, 133.25, 127.61, 125.21, 116.36, 115.01, 56.04, and 26.34 (Figure S21); and HRMS (ESI^+^), *m*/*z* 353.1373 [M+H]^+^ (found); *m*/*z* 353.1383 (calculated for [C_21_H_20_O_5_+H]^+^) (Figure S22).

##### 2,6-Bis((E)-4-hydroxy-3-methoxybenzylidene)cyclohexan-1-one (**7**)

The product was isolated as a yellow solid weighing 656 mg (1.8 mmol, 72% yield). The mp was 178 °C (Lit., 178–179 °C) [89].

Data were as follows: NMR ¹H (500 MHz, DMSO-*d_6_*) δ (ppm), 9.55 (s, 2H), 7.58 (s, 2H), 7.13 (sl, 2H), 7.05 (d, 2H, J = 10.0 Hz), 6.87 (d, 2H J = 10.0 Hz), 3.83 (s, 6H), 2.91 (t, 4H), and 1.74 (sl, 2H) (Figure S23); ^13^C NMR (DEPTQ) (125 MHz, DMSO-*d_6_*) δ, 188.95, 148.29, 147.90, 136.63, 127.39, 124.69, 116.01, 115.27, 56.10, 28.43, and 23.05 (Figure S24); HRMS (ESI^+^), *m*/*z* 367.1531 [M+H]^+^ (found); *m*/*z* 367.1540 (calculated for [C_22_H_22_O_5_+H]^+^) (Figure S25).

##### (3E,5E)-3,5-Bis(4-hydroxy-3-methoxybenzylidene)tetrahydro-4H-pyran-4-one (**8**)

The product was isolated as a green-yellow solid weighing 588.8 mg (1.6 mmol, 64% yield). The mp was 198–200 °C (Lit., 196–198 °C) [90].

Data were as follows: ^1^H NMR (500 MHz, DMSO-*d_6_*) δ, 3.82 (s, 6H), 4.90 (s, 4H), 6.86–6.89 (m, 4H), 7.02 (s, 2H), 7.60 (s, 2H), and 9.70 (s, 2H) (Figure S26); ^13^C NMR (DEPTQ) (125 MHz, DMSO-*d_6_*) δ, 55.65, 67.80, 114.93, 115.75, 124.62, 125.83, 130.82, 135.34, 147.62, 148.54, and 184.41 (Figure S27); HRMS (ESI^+^), *m*/*z* 369.1331 [M+H]^+^ (found); *m*/*z* 369.1332 (calculated for [C_21_H_20_O_6_+H]^+^) (Figure S28).

##### (3E,5E)-3,5-Bis(4-hydroxy-3-methoxybenzylidene)-1-methylpiperidin-4-one (**9**)

The product was isolated as an orange solid weighing 219.1 mg (0.58 mmol, 23% yield). The mp was 199–200 °C (Lit., 198–200 °C) [91].

Data were as follows: ^1^H NMR (500 MHz, DMSO-*d_6_*) δ, 2.40 (s, 3H), 3.71 (s, 4H), 3.81 (s, 6H), 6.87 (d, 2H, *J* = 8.3 Hz), 6.95 (dd, 2H, *J* = 8.3, 1.5 Hz), 7.07 (d, 2H, *J* = 1.5 Hz), 7.53 (s, 2H), and 9.64 (s, 2H) (Figure S29); ^13^C NMR (DEPTQ) (125 MHz, DMSO-*d_6_*) δ, 45.48, 55.67, 56.62, 115.06, 115.72, 124.20, 126.26, 131.00, 135.03, 147.56, 148.21, and 186.01 (Figure S30); HRMS (ESI^+^), *m*/*z* 382.1660 ([M+H]^+^ found); *m*/*z* 382.1649 (calculated for [C_22_H_23_NO_5_+H]^+^) (Figure S31).

##### (3Z,5Z)-3,5-Bis(4-hydroxy-3-methoxybenzylidene)dihydro-2H-thiopyran-4(3H)-one (**10**)

The product was isolated as a green solid weighing 921.6 mg (2.4 mmol, 96% yield). The mp was 97–98 °C (Lit., 98–100 °C) [92].

Data were as follows: ^1^H NMR (500 MHz, DMSO-*d_6_*) δ, 3.80 (s, 6H), 3.98 (s, 4H), 6.85 (d, 2H, *J* = 8.2 Hz), 7.00 (dd, 2H, *J* = 8.2, 1.6 Hz), 7.08 (d, 2H, *J* = 1.6 Hz), 7.54 (s, 2H), and 9.67 (s, 2H) (Figure S32); ^13^C NMR (DEPTQ) (125 MHz, DMSO-*d_6_*) δ, 29.65, 55.81, 114.73, 115.76, 124.38, 126.23, 131.71, 136.05, 147.70, 148.20, and 187.95 (Figure S33); HRMS (ESI^+^), *m*/*z* 385.1114 ([M+H]^+^ found); *m*/*z* 385.1104 (calculated for [C_21_H_20_O_5_S+H]^+^) (Figure S34).

##### Synthesis of (3Z,5Z)-3,5-Bis(4-hydroxy-3-methoxybenzylidene)dihydro-2H-thiopyran-4(3H)-one 1-oxide (**11**)

Product **10** (30 mg, 0.08 mmol) and hydrogen peroxide 35% (28 μL, 0.32 mmol) were dissolved in acetic acid (1 mL). After 30 min, the complete consumption of **10** was confirmed by TLC, and the reaction was poured into a 50 mL beaker containing crushed ice. The precipitate was then vacuum-filtered and dried at room temperature.

The product was isolated as a yellow solid weighing 32.0 mg (0.08 mmol, 100% yield). The mp was 180 °C (decomposition).

Data were as follows: ^1^H NMR (500 MHz, DMSO-*d_6_*) δ, 3.83 (s, 6H), 4.22 (d, 2H, *J* = 15.0 Hz), 4.42 (d, 2H, *J* = 15.0 Hz), 6.87 (d, 2H, *J* = 8.2 Hz), 7.03 (d, 2H, *J* = 8.2 Hz), 7.12 (s, 2H), 7.86 (s, 2H), and 9.67 (brs, 2H) (Figure S35); ^13^C NMR (DEPTQ) (125 MHz, DMSO-*d_6_*) δ, 48.12, 55.69, 114.53, 115.66, 123.88, 124.07, 125.73, 142.82, 147.60, 148.45, and 185.74 (Figure S36); HRMS (ESI^−^), *m*/*z* 399.0913 [M−H]^-^ (found); *m*/*z* 399.0907 (calculated for [C_21_H_20_O_6_S−H]^−^) (Figure S37).

##### Synthesis of (3Z,5Z)-3,5-Bis(4-hydroxy-3-methoxybenzylidene)dihydro-2H-thiopyran-4(3H)-one 1,1-dioxide (**12**)

Product **10** (39 mg, 0.1 mmol), OXONE^®^ (245 mg, 0.8 mmol), and diethylamine (58 μL, 0.5 mmol) were dissolved in 2 mL of an acetonitrile/water 1:3 mixture. After 12 h, the consumption of **10** was confirmed by TLC, and the reaction was poured into a 50 mL beaker containing crushed ice. The precipitate was then vacuum-filtered and dried at room temperature.

The product was isolated as an orange solid weighing 32.0 mg (0.08 mmol, 80% yield). The mp was 205 °C (decomposition).

Data were as follows: ^1^H NMR (500 MHz, DMSO-*d_6_*) δ, 3.83 (s, 6H), 4.68 (s, 4H), 6.89 (d, 2H, *J* = 7.9 Hz), 7.03 (d, 2H, *J* = 7.9 Hz), 7.13 (s, 2H), 7.82 (s, 2H), and 9.78 (s, 2H) (Figure S38); ^13^C NMR (DEPTQ) (125 MHz, DMSO-*d_6_*) δ, 52.26, 55.72, 114.77, 115.77, 124.29, 124.97, 125.01, 142.18, 147.73, 148.92, and 184.07 (Figure S39); HRMS (ESI^−^), *m*/*z* 415.0860 [M−H]^−^ (found); *m*/*z* 415.0857 (calculated for [C_21_H_20_O_7_S−H]^−^) (Figure S40).

#### 3.1.4. Stability Study of CUR **1** and Bis-Chalcones **4** and **8** by HPLC-DAD

The liquid chromatography spectrometry (LC-PDA) analyses were conducted on a Shimadzu LCMS 2020 (Shimadzu Inc., Kyoto, Japan). The analytical conditions were as follows: Column Restek, Allure Organic Acid C18, 150 mm × 4.6 mm × 5 µm (Bellefonte, PA, USA); mobile phase, 40% water with 0.1% formic acid, 60% acetonitrile with 0.1% formic acid, 1.0 mL/min, isocratic elution; injection volume, 5 µL; detectors, PDA (200–800 nm). Aliquots of 20 µL of 5 mM **1**, **4**, and **8** (dissolved in methanol) were added to 980 µL of PBS (137 mM NaCl, 10 mM phosphate, 2.7 mM KCl; pH 7.4). Samples were incubated at 37 °C for indicated times (0, 15 and 30 min, and 1 and 24 h). After incubation, 100 µL of reaction mixtures were added to 900 µL of HPLC mobile phase and injected [68].

### 3.2. Biological Assays

Mammalian cells were used to evaluate the cytotoxicity of the tested compounds. LLC-MK2 cells (ATCC^®^, Manassas, VA, USA) were used and cultured in Dulbecco’s modified Eagle’s medium (DMEM) supplemented with 5% fetal bovine serum (FBS), being incubated at 37 °C (5% CO_2_), with successive passages every 4–5 days. Cells were dissociated from the monolayer by treatment with a solution containing 0.25% *w*/*v* trypsin and 0.04% EDTA.

The antiparasitic activity assays used the Tulahuen C2C4-*LacZ* strain of the *Trypanosoma cruzi* parasite, being evaluated in the amastigote forms. Parasites of the morphologic form amastigotes and trypomastigotes were cultivated by successive reinfections in a monolayer of LLC-MK2 cells in DMEM medium + 2% FBS, being incubated at 37 °C (5% CO_2_). Trypomastigote forms were collected from the culture supernatant between the 5th and 10th day after infection and separated from non-adherent cells by differential centrifugation. Epimastigote evolutionary form parasites were cultivated in LIT medium (Liver Infusion Tryptose), supplemented with hemin (25 mg/L) + 10% FBS, and incubated at 28 °C, performing successive passages every 6–7 days. 

#### 3.2.1. Evaluation of Cytotoxicity Against LLC-MK2, A549, H460, and Human Peripheral Blood Mononuclear Cells

In a 96-well transparent plate, a suspension of 1 × 10^4^ LLC-MK2 cells (ATCC) in DMEM medium + 2% FBS was added. Cells were incubated at 37 °C (5% CO_2_) for 20 h and then washed with PBS to remove non-adherent cells. Cells were treated with increasing concentrations of the hybrids in triplicates, pre-diluted in DMEM + 2% FBS, and incubated for 120 h. The toxic effect of the molecules was also evaluated on human lung cancer cell lines (A549 and H460), as well as on PBMC. In addition, we investigated the effect of CIS, a first-line chemotherapeutic agent for the treatment of lung cancer, on the two human tumor cell lines. PBMC samples were obtained from buffy coats from various donors (Hospital Universitário Clementino Fraga Filho Blood Bank. Rio de Janeiro, RJ, Brazil) and isolated by gradient centrifugation using Ficoll–Paque PLUS (GE Healthcare, Arlington Heights, IL, USA) as previously described [93]. After isolation, the healthy cells, as well as the lung cancer cells, were treated with increasing concentrations (0.195–200 µM) of the curcuminoids. To assess the effect of CIS on the tumor cell lines, the cells were also exposed to increasing concentrations of the drug (0.097–25 µM). Similarly, LLC-MK2 cells were treated with increasing concentrations (2.56–200 µM) of the test compounds. In all experiments, untreated controls, vehicle (0.2% *v*/*v* DMSO), and blanks (no added cells) were included. After incubation (120 h for LLC-MK2 cells and 72 h for PBMC, A549, and H460 cells), the supernatant was removed, and the cell monolayer was washed with PBS, then the culture medium was renewed. Then, 20 µL of 3.0 mM MTT saline was added and incubated for another 1.5 h. The supernatant was removed, and the MTT formazan crystals were dissolved by adding 120 µL/well of DMSO. After incubation for 1.5 h to dissolve the MTT crystals away from light and at 37 °C, the absorbance was measured at λ = 570 nm using a plate reader.

#### 3.2.2. Evaluation of Trypanocidal Activity Against Amastigote Forms of *T. cruzi*

In a 96-well transparent plate, a suspension of 1 × 10^4^ LLC-MK2 cells (ATCC) in DMEM medium + 2% FBS was added. Cells were incubated at 37 °C (5% CO_2_) for adhesion for 3 h and then washed with PBS to remove non-adherent cells. A suspension containing 1.5 × 10^5^ trypomastigotes of the *T. cruzi* Tulahuen C2C4 *LacZ* strain was added to the cells, followed by incubation at 37 °C (5% CO_2_) for 20 h to establish infection. Non-internalized parasites were removed by three successive washes with PBS, followed by treatment with increasing concentrations of the hybrids (0.64–100 µM) in triplicates, pre-diluted in DMEM + 2% FBS. Untreated vehicle (0.2% *v*/*v* DMSO) and blank (no added parasites) controls were included in the experiment. Increasing concentrations of Benznidazole were used as the positive control. After incubation for 5 days (120 h), 30 µL of 0.5 mM chlorophenol red substrate *β*-galactopyranoside (CPRG) in PBS was added with 0.9% *v*/*v* of Igepal CA-630. After incubation for 1.5 h, absorbance was measured at λ = 570 nm using a plate reader [83].

#### 3.2.3. Cell Cycle Arrest of A549 Cells After Treatment with Curcuminoids

The cell cycle phase was determined using a PI solution, according to a method described elsewhere [94]. For the assay, cells were plated at a density of 1 × 10^6^ cells/well in six-well plates. After treatment for 72 h with the most active curcuminoids (8, 11, and 12), the cells were centrifuged at 200× *g* for 7 min, the supernatant was discarded, and the precipitate was resuspended in HBSS plus PI Solution (50 μg/mL, RNAse 1 mg/mL and Triton X-100 0.2%) for 30 min at 4 °C. Cell cycle determination was performed on a FACScalibur flow cytometer (BD Biosciences, San Diego, CA, USA). Post-analysis of the cell cycle assay was performed using Summit software (version 4.3, Dako Colorado, Fort Collins, CO, USA).

#### 3.2.4. Cell Death Analysis by Flow Cytometry

Cell death analysis was performed by using an Annexin V/PI double staining kit (PharMingen, San Diego, CA, USA) as previously described [95]. For the assay, the cells were plated at a density of 1 × 10^6^ cells/well in six-well plates, 2 mL/well. After treatment for 48 h with the most active curcuminoid (**12**), A549 cells were centrifuged at 200× *g* for 5 min, the supernatant was discarded, and the precipitate was suspended in 100 µL of a solution containing Annexin V-FITC and propidium iodide (PI). After incubating for 15 min at room temperature in the dark, 400 µL of binding buffer was added. The two-color flow cytometry analysis was performed on a FACSCalibur device (Becton Dickinson, Franklin Lakes, NJ, USA). Post-analysis was performed in Summit software (version 4.3, Dako Colorado, Fort Collins, CO, USA).

#### 3.2.5. ADME Prediction

The absorption, distribution, metabolization, and excretion (ADME); physicochemical; drug-like; and related parameters were estimated for curcuminoids **1**–**12** with the free web server SwissADME (version 2025, http://www.swissadme.ch/index.php, accessed on 22 January 2025) [74,96].

#### 3.2.6. Molecular Docking Calculations

The structures of the cancer-related targets and the three isoforms of cytochrome P450 (CYP450) were obtained in the Protein Data Bank (PDB) with the access codes 1M17 [97], 2W3L [98], 3DCY, 3G0E [79], 6QHD [99], 2HI4 [100], 1R9O [101], and 1W0F [102], while the three-dimensional structures for compounds **1**–**12** were built and energy-minimized with Spartan’18 software (Wavefunction, Inc., Irvine, CA, USA) [103]. The molecular docking calculations were performed with GOLD 2024.3 software (Cambridge Crystallographic Data Centre, Cambridge, UK) [104], considering an 8Å radius around the active site. Redocking studies were carried out on the inhibitors sunitinib and erlotinib crystallized into the active sites of 3G0E and 1M17, respectively. In this case, the lowest root mean square deviation (RMSD) value was obtained from ChemPLP. Thus, this standard function was used as the scoring function. The Protein-Ligand Interaction Profiler (PLIP) [105] was applied to identify the main amino acid residues, while three-dimensional representations were generated using PyMOL Molecular Graphics System 1.0 level software (Delano Scientific LLC software, Schrodinger, New York, NY, USA) [106].

#### 3.2.7. Statistical Analysis

The IC_50_ (the concentration of the drug required for 50% inhibition of cell proliferation) and the LD_50_ (the lethal dose that kills 50% of the cells) were calculated using nonlinear regression with GraphPad Prism version 5.0 software (GraphPad Software, San Diego, CA, USA).

## 4. Conclusions

The molecular simplification analogs of curcumin were designed and obtained through a simple synthetic strategy, generating the desired molecules in reasonable to good yields. The antitumor activity assays against A549 and H460 lung cancer cell lines validated our molecular design strategy since some of the obtained bis-chalcones showed higher potency than the natural diaryleptanoids and higher selectivity indices. The pharmacokinetic property in silico predictions demonstrated the active derivatives’ excellent druggability profile. Additionally, molecular modeling calculations on some of the main biochemical targets involved in the modulation of tumor cell proliferation showed interesting results, with special emphasis on p53 (tumor suppressor gene, involved in several cellular mechanisms, including DNA repair, apoptosis, and cell cycle arrest) and the KIT enzyme, suggesting these two proteins as the main biochemical targets. These theoretical results were reinforced by the correlation between the docking score values for four of the bis chalcones (**4**, **8**, **11**, and **12**), which also presented the best experimental IC_50_ values in the in vitro assays. The results obtained in the evaluation of the activity of the set of natural diarylheptanoids (**1**–**3**), as well as the collection of molecular simplification synthetic derivatives (**4**–**12**), against intracellular amastigotes of *T. cruzi* demonstrated the potential of these substances in the development of new antiparasitic drugs.

## Figures and Tables

**Table 1:** Antiproliferative activity of natural curcuminoids (**1**–**3**) and simplified analogs (bis-chalcones **4** and **6**–**12** and chalcone **5**) against A549 and H460 tumor cell lines and cytotoxicity on peripheral blood mononuclear cells.

Compounds	A549	H460	PBMC	Selective Index SI A549/SI H460
	IC_50_ (µM)		LD_50_ (µM)	
**1 CUR**	56.48 ± 3.75	37.85 ± 8.81	146.5 ± 12.08	2.59/3.87
**2 DMC**	73.29 ± 6.98	25.05 ± 5.55	144.3 ± 12.30	1.96/5.76
**3 BDMC**	46.52 ± 3.45	27.09 ± 4.35	134.1 ± 13.30	2.88/13.14
**4**	18.05 ± 4.72	10.20 ± 3.76	>200	11.08/19.60
**5**	58.02 ± 6.75	1.74 ± 0.28	119.1 ± 16.35	2.05/68.44
**6**	71.28 ± 1.53	36.14 ± 7.14	141.1 ± 18.53	1.97/3.90
**7**	76.16 ± 9.49	42.57 ± 3.94	>200	2.62/4.69
**8**	8.10 ± 1.27	1.46 ± 0.28	131.8 ± 14.52	16.27/82.37
**9**	61.11 ± 6.20	9.89 ± 3.03	73.25 ± 5.87	1.19/7.40
**10**	36.97 ± 8.28	12.8 ± 3.94	186.4 ± 35.15	5.04/14.56
**11**	7.74 ± 1.65	1.02 ± 0.29	>200	25.97/196.07
**12**	5.89 ± 2.81	0.27 ± 0.20	173.0 ± 37.4	29.37/640.74
**Cisplatin** *	6.05 ± 0.58	2.96 ± 0.44	n.t.	n.c./n.c.

* Reference drug; n.t., not tested; n.c., not calculated.

**Table 2:** Selected physicochemical, drug-likeness, and pharmacokinetic properties predicted by the free web server SwissADME.

Compound	Consensus Log P_o/w_^a^	CYP Inhibition (Isoform)		Drug-Likeness (Number of Violations)				
			TPSA (Å^2^)	Lipinski	Ghose	Veber	Egan	Muegge
**1**	3.03	Yes (2C9, 3A4)	93.06	Yes	Yes	Yes	Yes	Yes
**2**	3.00	Yes (1A2, 2C9, 3A4)	83.83	Yes	Yes	Yes	Yes	Yes
**3**	2.83	Yes (1A2, 2C9, 3A4)	74.60	Yes	Yes	Yes	Yes	Yes
**4**	3.05	Yes (1A2, 2C9, 3A4)	75.99	Yes	Yes	Yes	Yes	Yes
**5**	2.63	Yes (1A2, 2C9, 3A4)	75.99	Yes	Yes	Yes	Yes	Yes
**6**	2.63	Yes (1A2, 2C9, 3A4)	75.99	Yes	Yes	Yes	Yes	Yes
**7**	2.63	Yes (1A2, 2C9, 3A4)	75.99	Yes	Yes	Yes	Yes	Yes
**8**	2.63	Yes (1A2, 2C9, 3A4)	85.22	Yes	Yes	Yes	Yes	Yes
**9**	2.63	Yes (1A2, 2C9, 3A4)	79.23	Yes	Yes	Yes	Yes	Yes
**10**	2.63	Yes (1A2, 2C9, 3A4)	101.3	Yes	Yes	Yes	Yes	Yes
**11**	2.63	Yes (1A2, 2C9, 3A4)	112.3	Yes	Yes	Yes	Yes	Yes
**12**	2.63	Yes (1A2, 2C9, 3A4)	118.5	Yes	Yes	Yes	Yes	Yes

^a^ Consensus log P_o/w_: octanol/water partition coefficient calculated as the mean of five distinct predictive log P methods.

**Table 3:** Docking score values (dimensionless) for the interaction between curcuminoids **1**–**12** and EGFR (PDB code 1M17), BCL2 (PDB code 2W3L), p53 (PDB code 3DCY), KIT kinase domain (PDB code 3G0E), and STAT3 (PDB code 6QHD).

Compound	1M17	2W3L	3DCY	3G0E	6QHD
**1**	64.6	62.6	56.1	60.1	60.3
**2**	66.7	62.4	55.3	58.2	62.9
**3**	61.1	58.7	53.6	58.7	59.6
**4**	69.2	56.3	67.9	69.4	55.2
**5**	60.5	57.0	66.7	70.0	51.6
**6**	69.0	57.2	58.0	57.5	49.0
**7**	67.7	55.6	56.4	58.5	45.3
**8**	69.5	50.4	69.8	66.6	48.9
**9**	59.9	49.4	59.3	67.3	53.8
**10**	55.6	54.9	59.1	65.1	55.1
**11**	68.3	53.5	66.8	69.7	53.3
**12**	69.6	50.0	69.9	70.1	54.1

**Table 4:** Trypanocidal activity of conformationally restricted diarylpentanoids (**8**–**12**) against intracellular *T. cruzi* amastigotes and cytotoxicity on LLC-MK2 host cells.

Compounds	*T. cruzi* Amastigotes (Tulahuen C2C4-*LacZ*) 120 h	LLC-MK2 (Host Cells) 120 h	Selective Index
	IC_50_ (µM)		
**1 CUR**	17.75 ± 6.88	25.69 ± 6.05	1.44
**2 DMC**	31.31 ± 0.77	22.69 ± 3.71	0.72
**3 BDMC**	17.61 ± 0.26	19.68 ± 0.87	1.11
**4**	3.92 ± 0.33	< 6.4	n.c.
**5**	15.64 ± 4.08	8.72 ± 2.42	0.55
**6**	8.35 ± 2.35	< 6.4	n.c.
**7**	4.91 ± 0.40	< 6.4	n.c.
**8**	12.78 ± 5.17	20.57 ± 4.65	1.60
**9**	9.60 ± 1.61	28.41 ± 3.80	2.95
**10**	5.87 ± 2.89	12.97 ± 2.36	2.20
**11**	16.70 ± 4.67	28.46 ± 5.17	1.70
**12**	7.33 ± 0.43	11.49 ± 3.71	1.56
Benznidazole *	1.50 ± 0.33	> 200	> 133

* Reference drug. n.c., not calculated.

## Data Availability

Data are contained within the article and the Supplementary Materials.

## Supplementary Materials

The following supporting information can be downloaded at https://www.mdpi.com/article/10.3390/ph18040456/s1: Figure S1: The BOILED-Egg graphic for the curcuminoids **1**–**12**. The HIA, BBB, PGP+, and PGP- are passive gastrointestinal absorption, blood-brain barrier permeation, P-glycoprotein1 substrate, and P-glycoprotein1 non-substrate, respectively. WLOGP and TPSA are indicators of lipophilicity and apparent polarity, respectively; Figure S2: Computed bioavailability radar for the compounds (A) **1**, (B) **2**, (C) **3**, (D) **4**, (E) **5**, (F) **6**, (G) **7**, (H) **8**, (I) **9**, (J) **10**, (K) **11**, and (L) **12**. LIPO, SIZE, POLAR, INSOLU, INSATU, and FLEX means lipophilicity, molecular weight, topological polar surface area (TPSA), solubility parameter (log S), unsaturated, and flexibility, respectively; Figure S3: Superposition of the best docking pose to **1**–**12** into the catalytic site of cytochrome P450 isoforms (A) 1A2, (B) 2C9, and (C) 3A4. Elements’ color: oxygen, nitrogen, and sulfur in red, dark blue, and yellow, respectively. To better visualize, hydrogen atoms were omitted; Figure S4: (**entry 1**) Chromatographic analysis of commercial curcumin shows the mixture of three main curcuminoids (**entry 2**). The three major curcuminoids from the rhizomes of *Curcuma longa* are separated by recrystallization and followed by open-column chromatography—in the sequence: (a) CUR, (b) DMC, (c) BDMC. The two thin-layer chromatographic analyses were performed on aluminum plates coated with silica-gel (0.25mm thick) using a 2% dichloromethane-methanol mixture as eluent. The spots were visualized under ultraviolet light (356 nm); Figure S5: ^1^H NMR spectrum (500 MHz, DMSO-*d*_6_) of CUR (**1**); Figure S6: DEPT-Q ^13^C NMR spectrum (125 MHz, DMSO-*d*_6_) of natural CUR (**1**); Figure S7: HRMS (Q-TOF) of CUR (**1**); Figure S8: ^1^H NMR spectrum (500 MHz, DMSO-*d*_6_) of DMC (**2**); Figure S9: DEPT-Q ^13^C NMR spectrum (125 MHz DMSO-*d*_6_) of DMC (**2**); Figure S10: HRMS (Q-TOF) of DMC (**2**); Figure S11: ^1^H NMR spectrum (500 MHz, DMSO-*d*_6_) of BDMC (**3**); Figure S12: DEPT-Q ^13^C NMR spectrum (125 MHz, DMSO-*d*_6_) of BDMC (**3**); Figure S13: HRMS (Q-TOF) of BDMC (**3**); Figure S14: ^1^H NMR spectrum (500 MHz, CDCl_3_/CD_3_OD) of chalcone (**5**); Figure S15: DEPT-Q ^13^C NMR spectrum (125 MHz, CDCl_3_/CD_3_OD) of chalcone (**5**); Figure S16: HRMS (Q-TOF) of chalcone (**5**); Figure S17: ^1^H NMR spectrum (500 MHz, CDCl_3_) of bis-chalcone (**4**); Figure S18: DEPT-Q ^13^C NMR spectrum (125 MHz, CDCl_3_) of bis-chalcone (**4**); Figure S19: HRMS (Q-TOF) of bis-chalcone (**4**); Figure S20: ^1^H NMR spectrum (500 MHz, DMSO-*d*_6_) of bis-chalcone (**6**); Figure S21: DEPT-Q ^13^C NMR spectrum (125 MHz, DMSO-*d*_6_) of bis-chalcone (**6**); Figure S22: HRMS (Q-TOF) of bis-chalcone (**6**); Figure S23: ^1^H NMR spectrum (500 MHz, DMSO-*d*_6_) of bis-chalcone (**7**); Figure S24: DEPT-Q ^13^C NMR spectrum (125 MHz, DMSO-*d*_6_) of bis-chalcone (**7**); Figure S25: HRMS (Q-TOF) of bis-chalcone (**7**); Figure S26: ^1^H NMR spectrum (500 MHz, DMSO-*d*_6_) of bis-chalcone (**8**); Figure S27: DEPT-Q ^13^C NMR spectrum (125 MHz, DMSO-*d*_6_) of bis-chalcone (**8**); Figure S28: HRMS (Q-TOF) of bis-chalcone (**8**); Figure S29: ^1^H NMR spectrum (500 MHz, DMSO-*d*_6_) of bis-chalcone (**9**); Figure S30: DEPT-Q ^13^C NMR spectrum (125 MHz, DMSO-*d*_6_) of bis-chalcone (**9**); Figure S31: HRMS (Q-TOF) of bis-chalcone (**9**); Figure S32: ^1^H NMR spectrum (500 MHz, DMSO-*d*_6_) of bis-chalcone (**10**); Figure S33: DEPT-Q ^13^C NMR spectrum (125 MHz, DMSO-*d*_6_) of bis-chalcone (**10**); Figure S34: HRMS (Q-TOF) of bis-chalcone (**10**); Figure S35: ^1^H NMR spectrum (500 MHz, DMSO-*d*_6_) of bis-chalcone (**11**); Figure S36: DEPT-Q ^13^C NMR spectrum (125 MHz, DMSO-*d*_6_) of bis-chalcone (**11**); Figure S37: HRMS (Q-TOF) of bis-chalcone (**11**); Figure S38: ^1^H NMR spectrum (500 MHz, DMSO-*d*_6_) of bis-chalcone (**12**); Figure S39: DEPT-Q ^13^C NMR spectrum (125 MHz, DMSO-*d*_6_) of bis-chalcone (**12**); Figure S40: HRMS (Q-TOF) of bis-chalcone (**12**).
