# Supplementary material for: Design and Synthesis of Bis-Chalcones as Curcumin Simplified Analogs and Assessment of Their Antiproliferative Activities Against Human Lung Cancer Cells and Trypanosoma cruzi Amastigotes
Source: Pharmaceuticals (Basel). 2025 Mar 24;18(4):456. doi: 10.3390/ph18040456 (PMC12030033; doi:10.3390/ph18040456)
Supplement: Supplementary file 1 [file pharmaceuticals-18-00456-s001.zip › pharmaceuticals-3512987-supplementary.pdf]

SUPPLEMENTAL MATERIAL

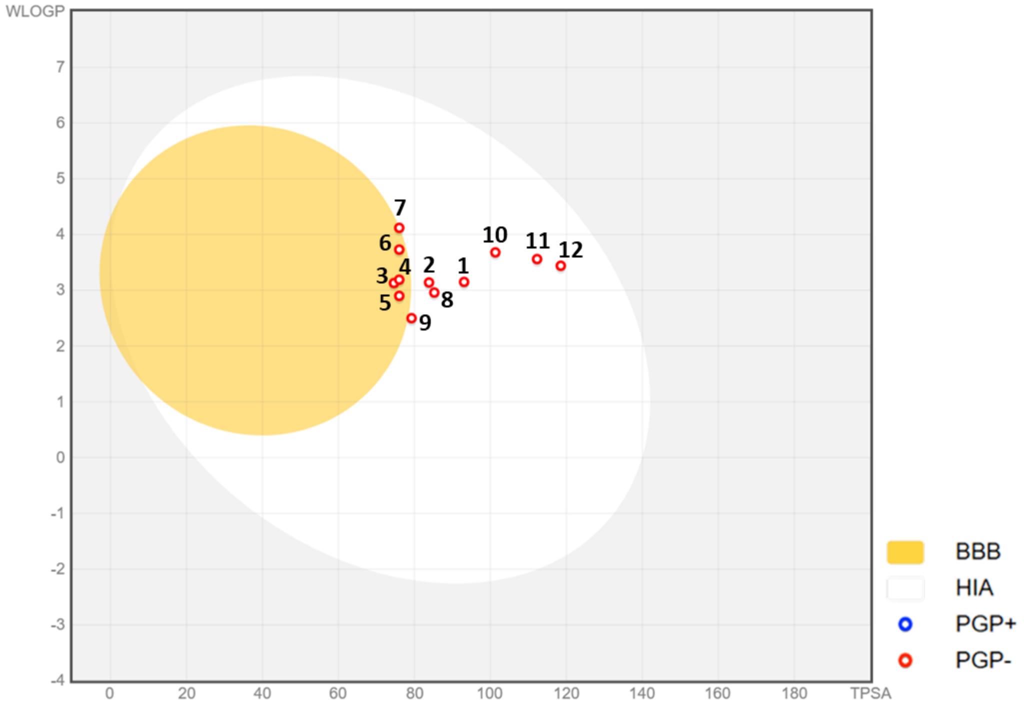

Figure S1. The BOILED-Egg graphic for the curcuminoids **1-12**. The HIA, BBB, PGP^+, and PGP^- are passive gastrointestinal absorption, blood-brain barrier permeation, P-glycoprotein1 substrate, and P-glycoprotein1 non-substrate, respectively. WLOGP and TPSA are indicators of lipophilicity and apparent polarity, respectively.

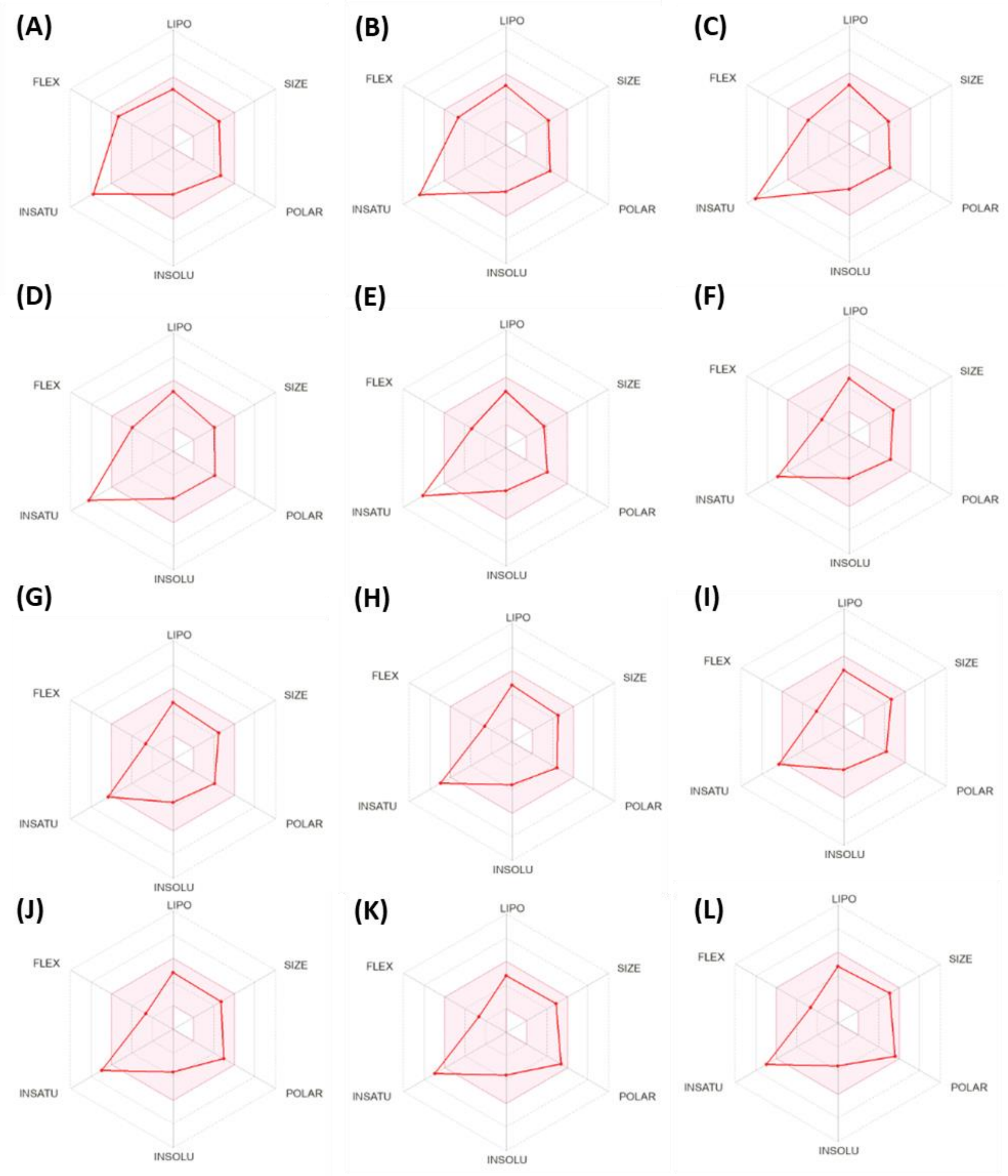

Figure S2. Computed bioavailability radar for the compounds (A) **1**, (B) **2**, (C) **3**, (D) **4**, (E) **5**, (F) **6**, (G) **7**, (H) **8**, (I) **9**, (J) **10**, (K) **11**, and (L) **12**. LIPO, SIZE, POLAR, INSOLU, INSATU, and FLEX means lipophilicity, molecular weight, topological polar surface area (TPSA), solubility parameter (log S), unsaturated, and flexibility, respectively.

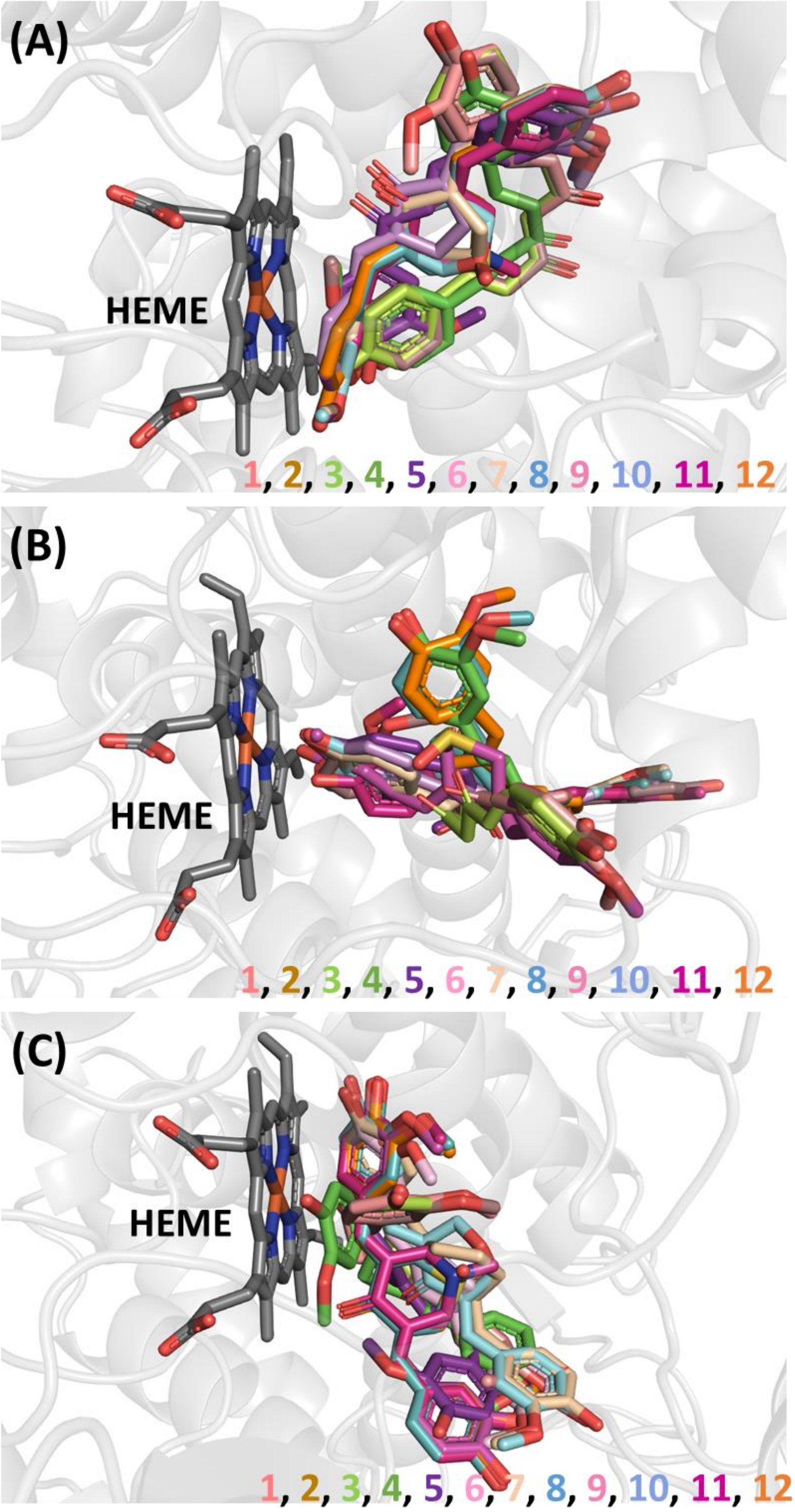

Figure S3. Superposition of the best docking pose to **1-12** into the catalytic site of cytochrome P450 isoforms (A) 1A2, (B) 2C9, and (C) 3A4. Elements' color: oxygen, nitrogen, and sulfur in red, dark blue, and yellow, respectively. To better visualize, hydrogen atoms were omitted.

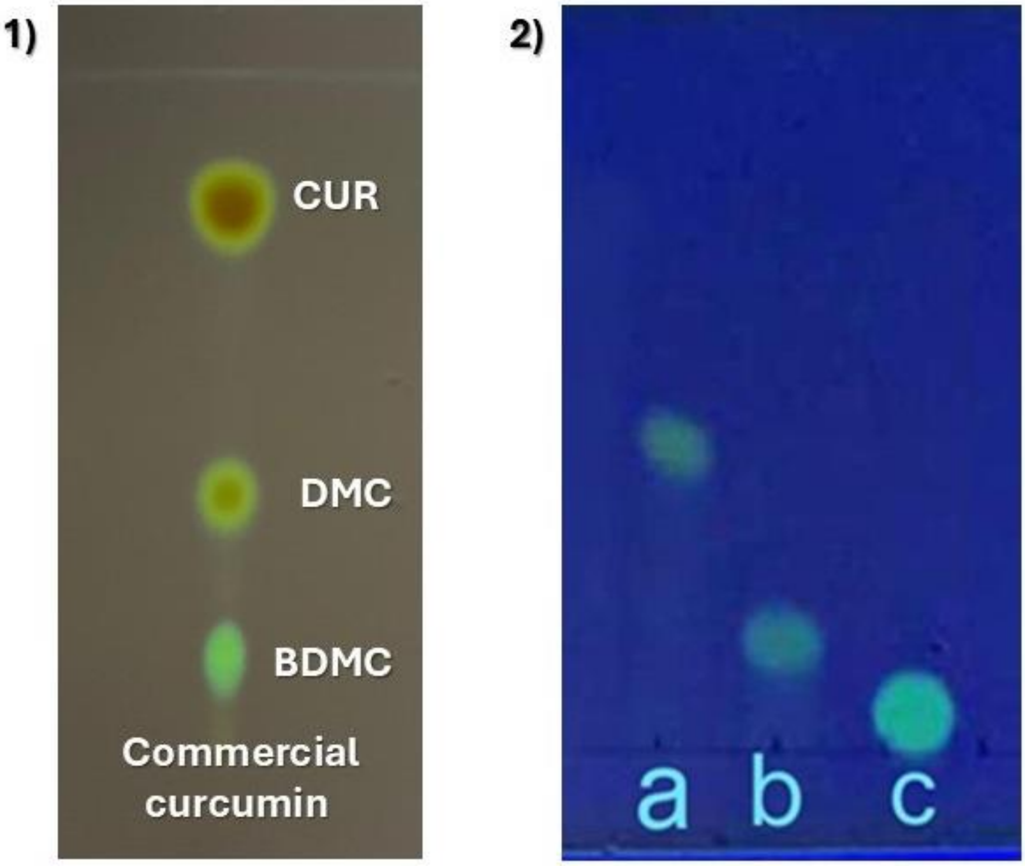

Figure S4. (**entry 1**) Chromatographic analysis of commercial curcumin shows the mixture of three main curcuminoids (**entry 2**). The three major curcuminoids from the rhizomes of *Curcuma longa* are separated by recrystallization and followed by open-column chromatography—in the sequence: (a) CUR, (b) DMC, (c) BDMC. The two thin-layer chromatographic analyses were performed on aluminum plates coated with silica-gel (0.25mm thick) using a 2% dichloromethane-methanol mixture as eluent. The spots were visualized under ultraviolet light (356 nm).

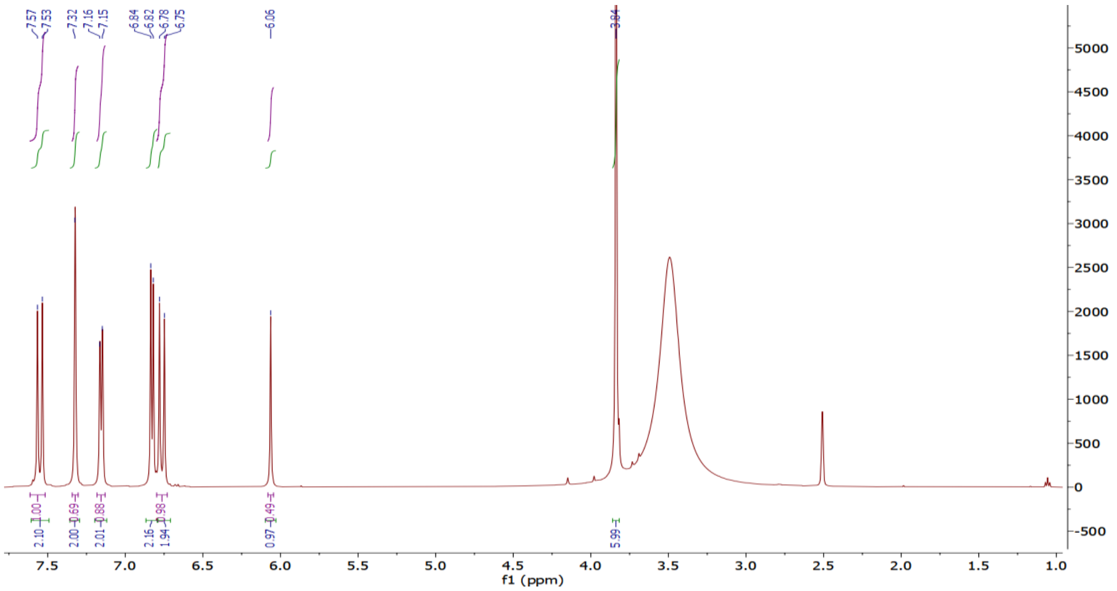

Figure S5. ^{1}H NMR spectrum (500 MHz, DMSO-d_6) of CUR (**1**).

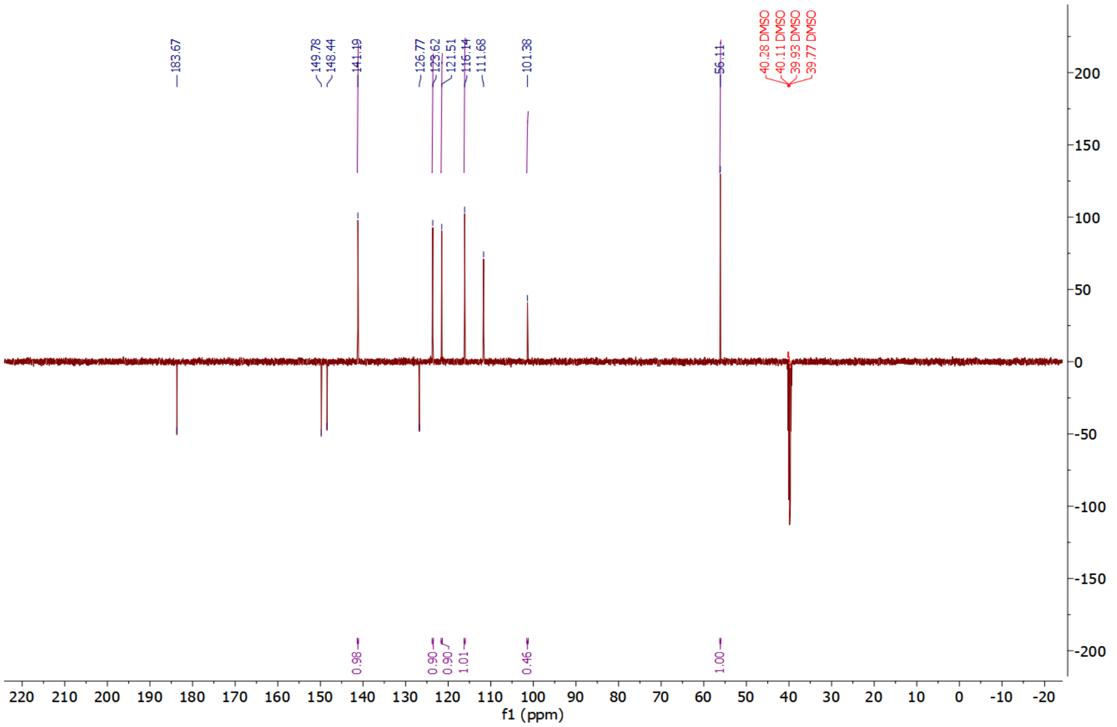

Figure S6. DEPT-Q ^{13}C NMR spectrum (125 MHz, DMSO-d_6) of natural CUR (**1**).

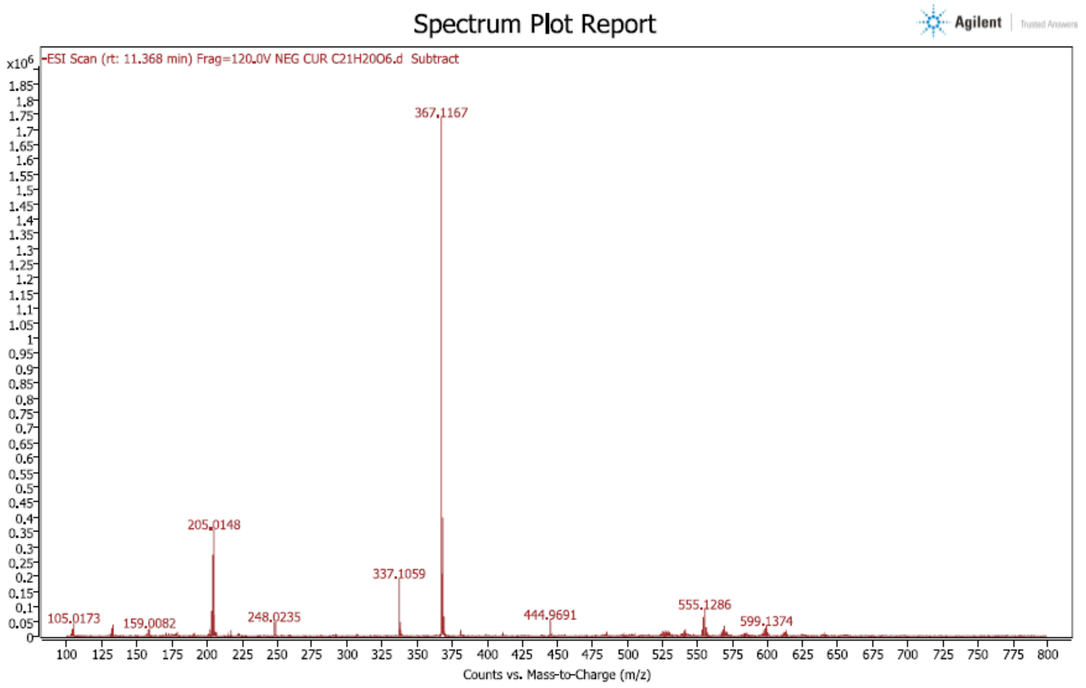

Figure S7. HRMS (Q-TOF) of CUR (**1**).

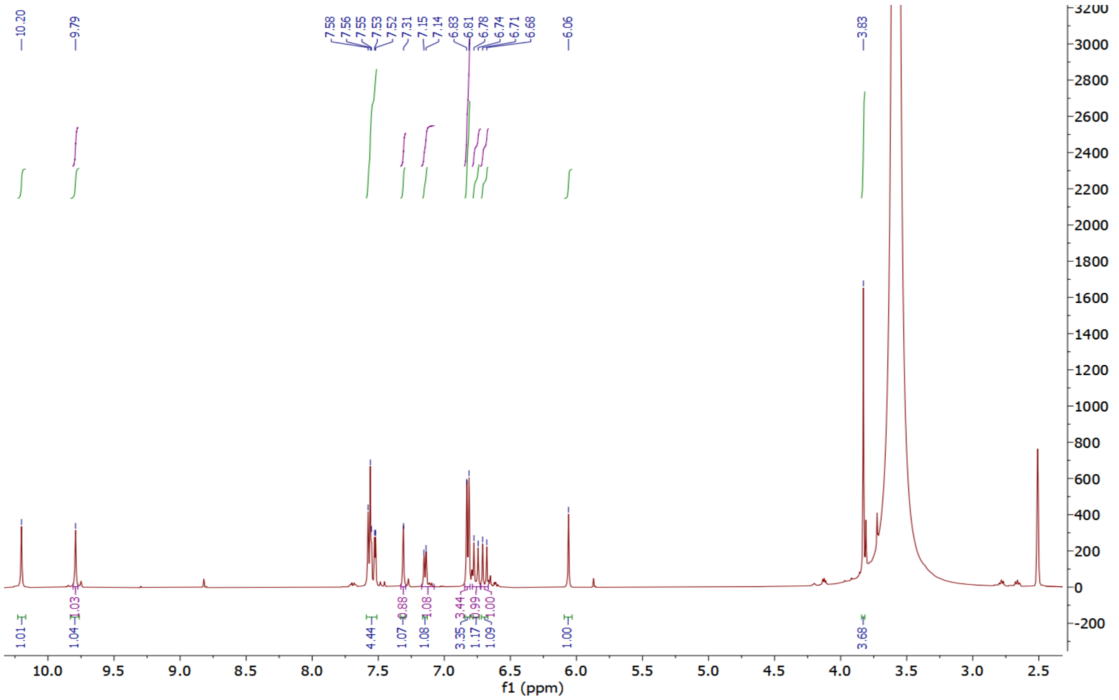

Figure S8. ^{1}H NMR spectrum (500 MHz, DMSO-d_6) of DMC (**2**).

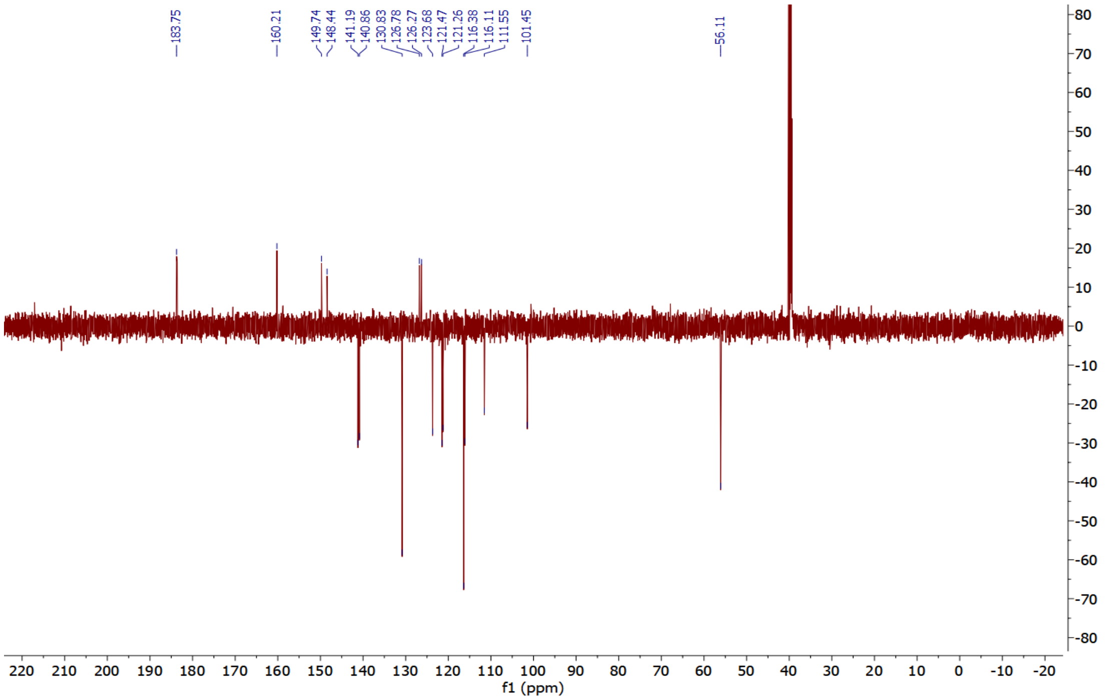

Figure S9. DEPT-Q ^{13}C NMR spectrum (125 MHz, DMSO-d_6) of DMC (**2**).

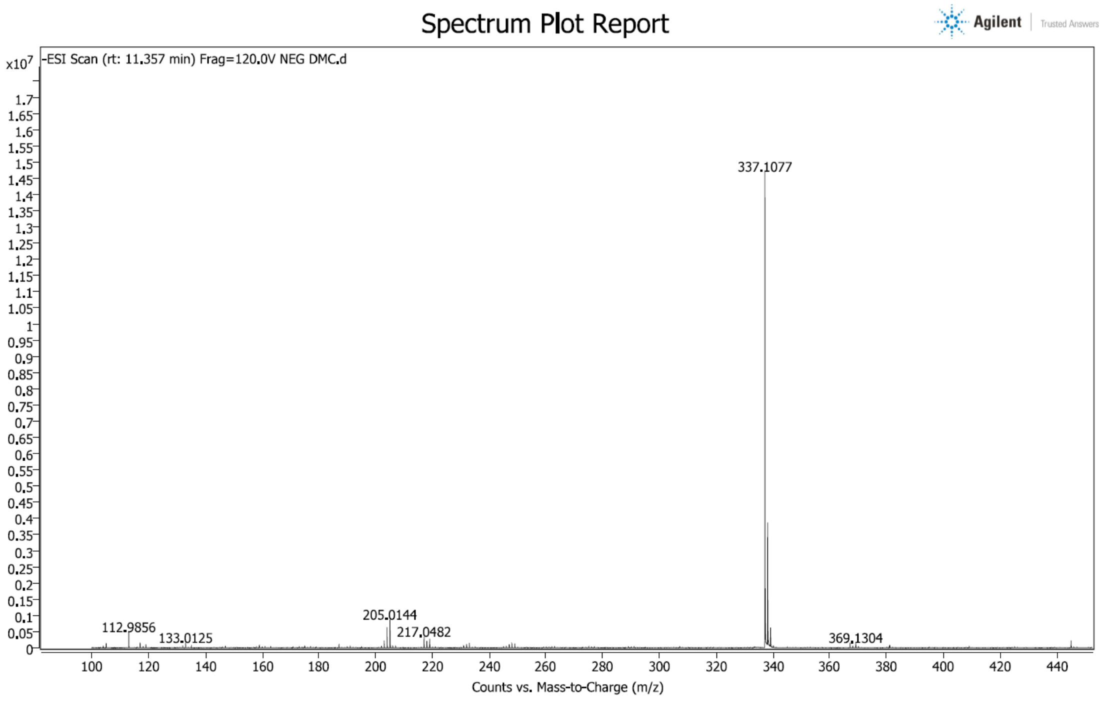

Figure S10. HRMS (Q-TOF) of DMC (**2**).

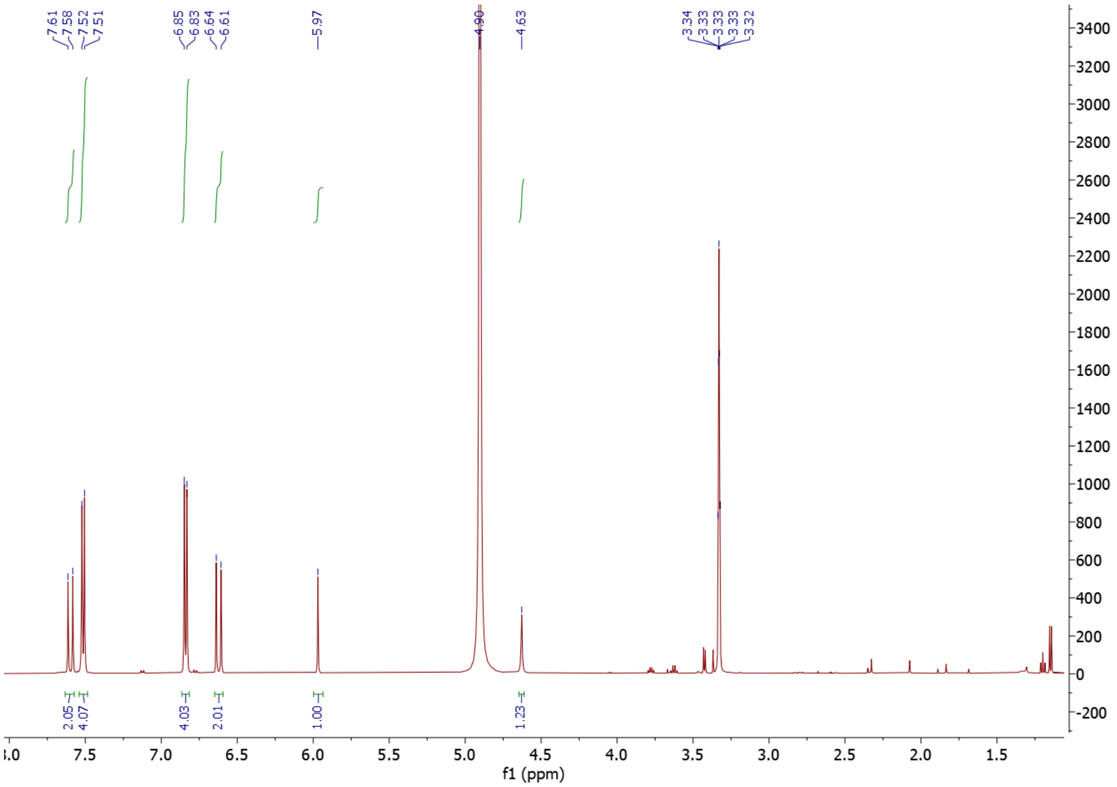

Figure S11. ^{1}H NMR spectrum (500 MHz, DMSO-d_6) of BDMC (**3**).

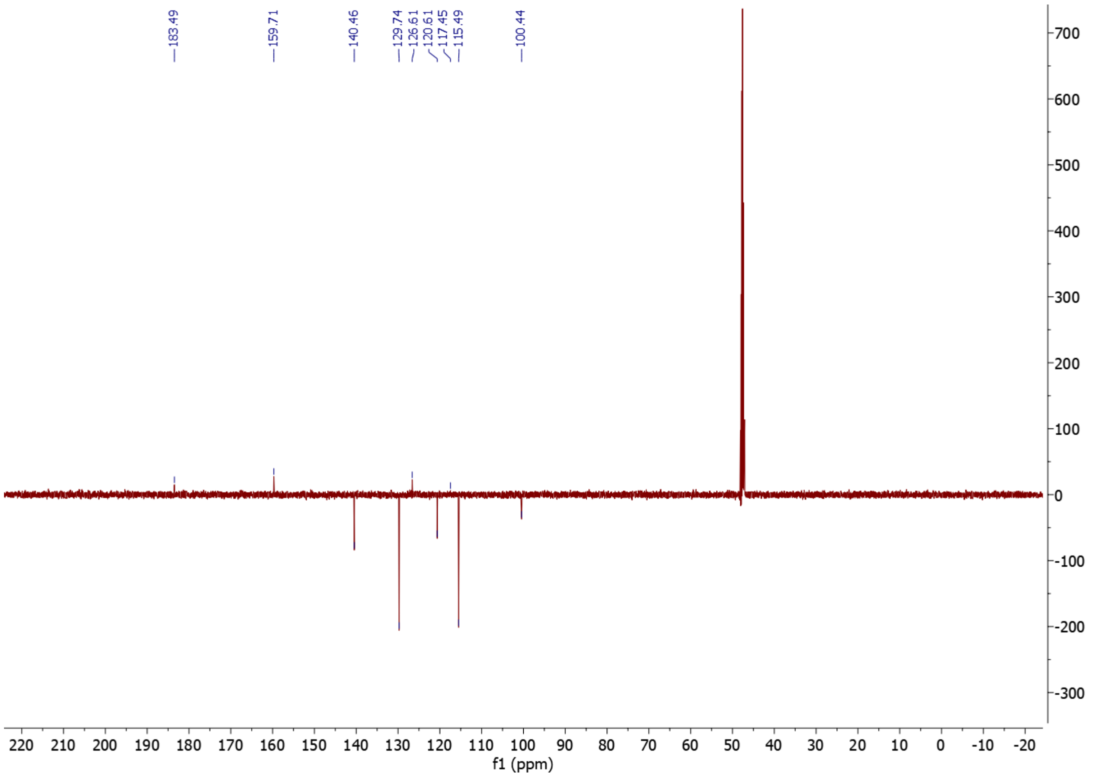

Figure S12. DEPT-Q ^{13}C NMR spectrum (125 MHz, DMSO-d_6) of BDMC (**3**).

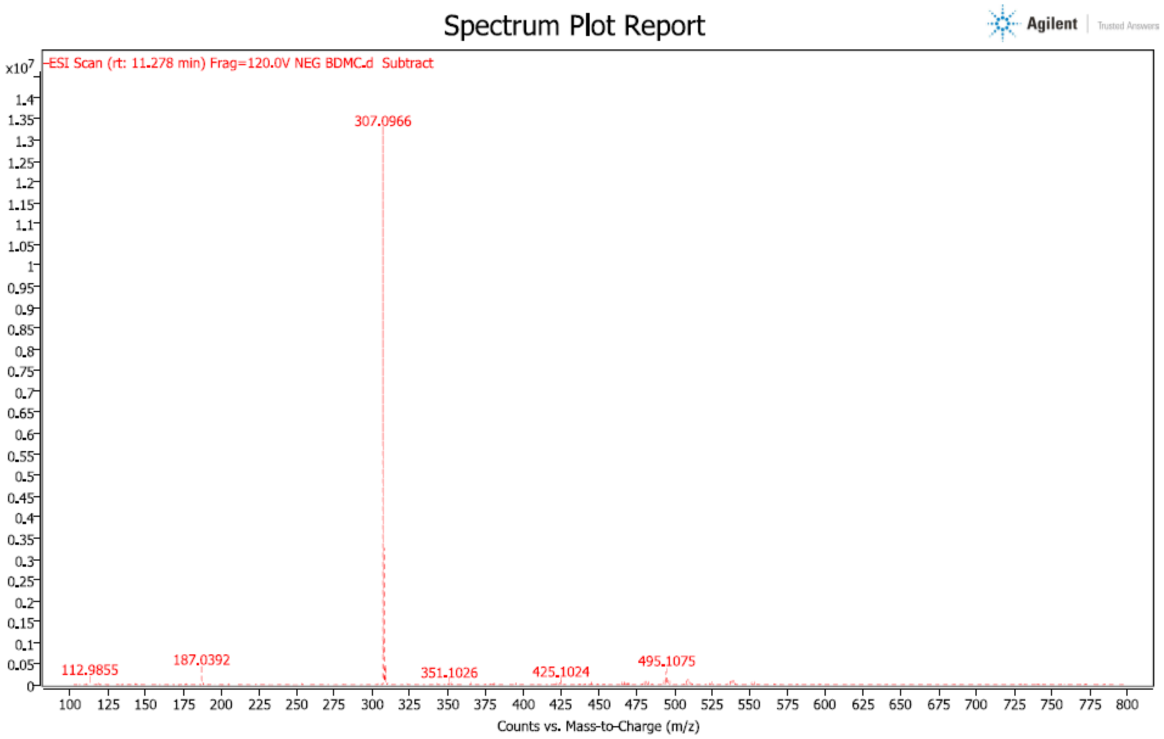

Figure S13. HRMS (Q-TOF) of BDMC (**3**).

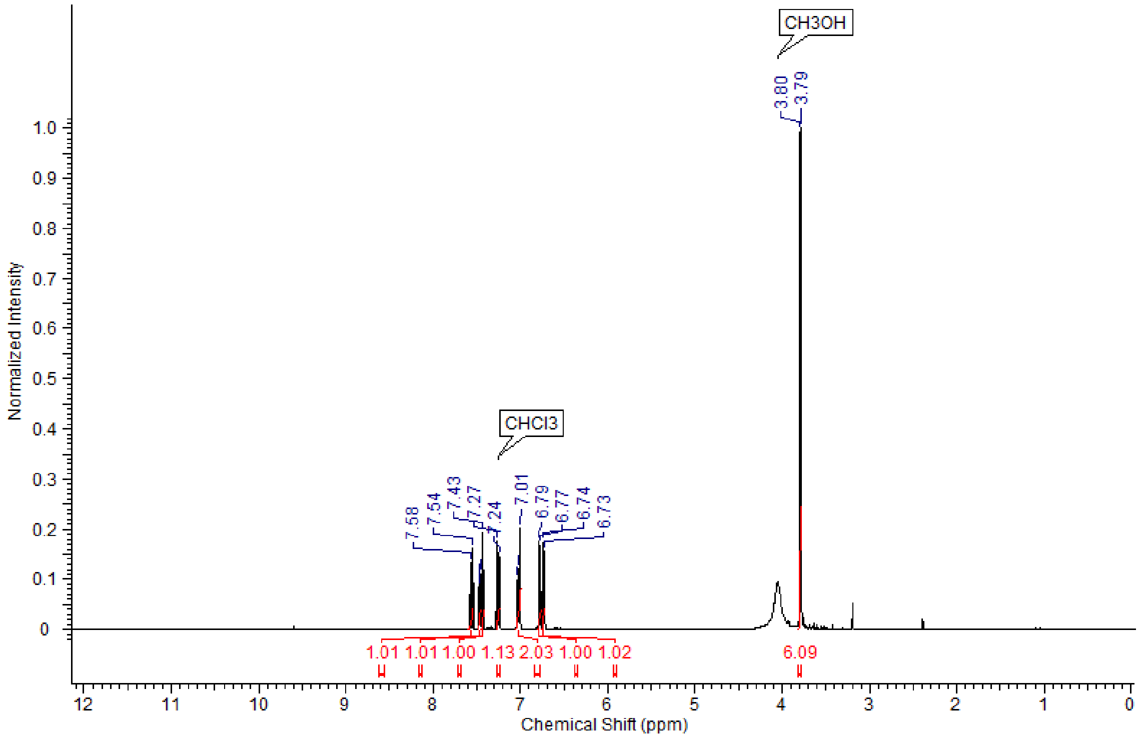

Figure S14. ¹H NMR spectrum (500 MHz, $CDCl_3/CD_3OD$) of chalcone (**5**).

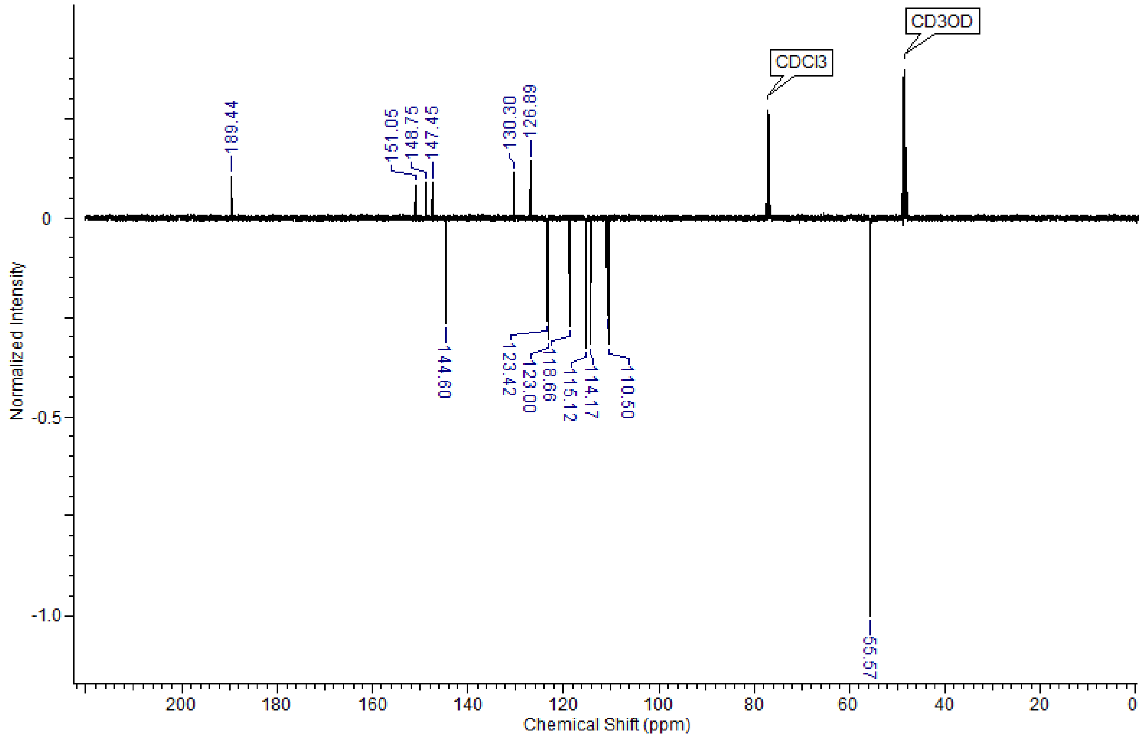

Figure S15. DEPT-Q ^{13}C NMR spectrum (125 MHz, $CDCl_3/CD_3OD$) of chalcone (**5**).

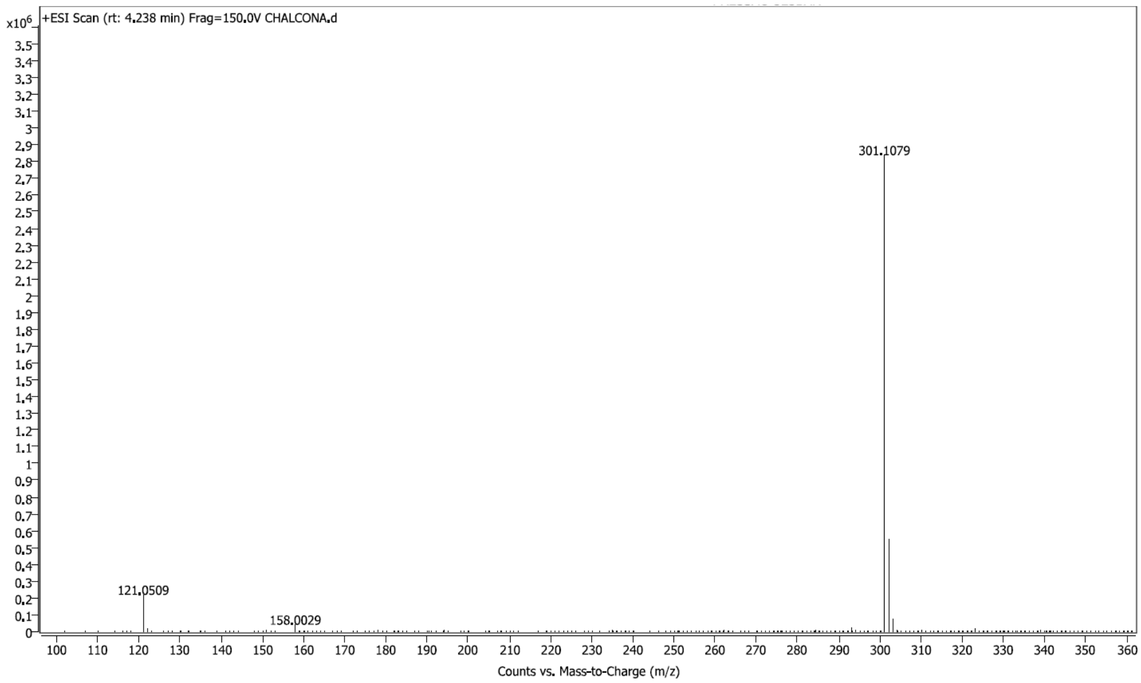

Figure S16. HRMS (Q-TOF) of chalcone (**5**).

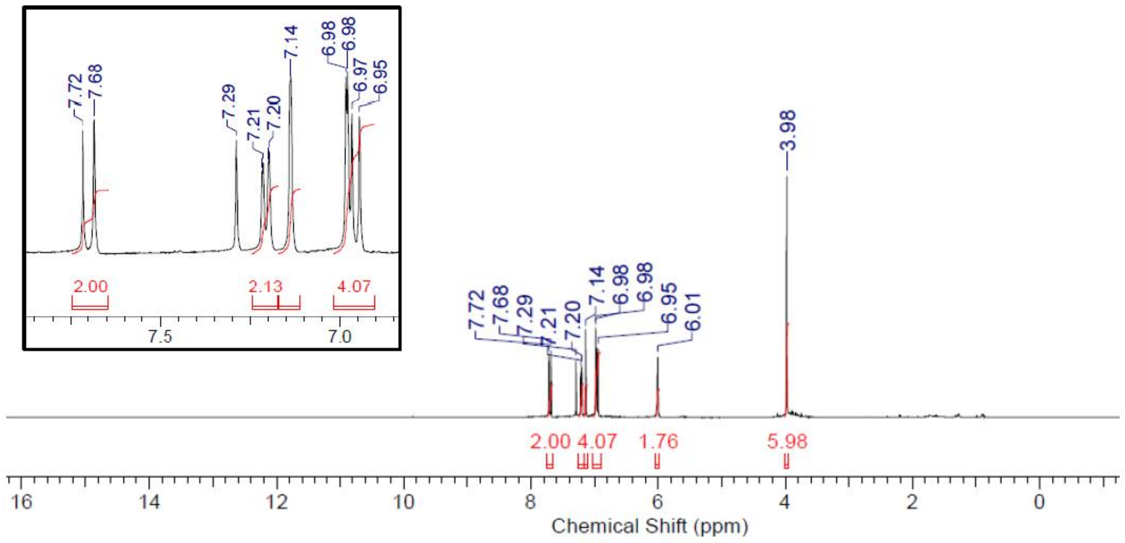

Figure S17, ^{1}H NMR spectrum (500 MHz, $CDCl_3$) of bis-chalcone (**4**).

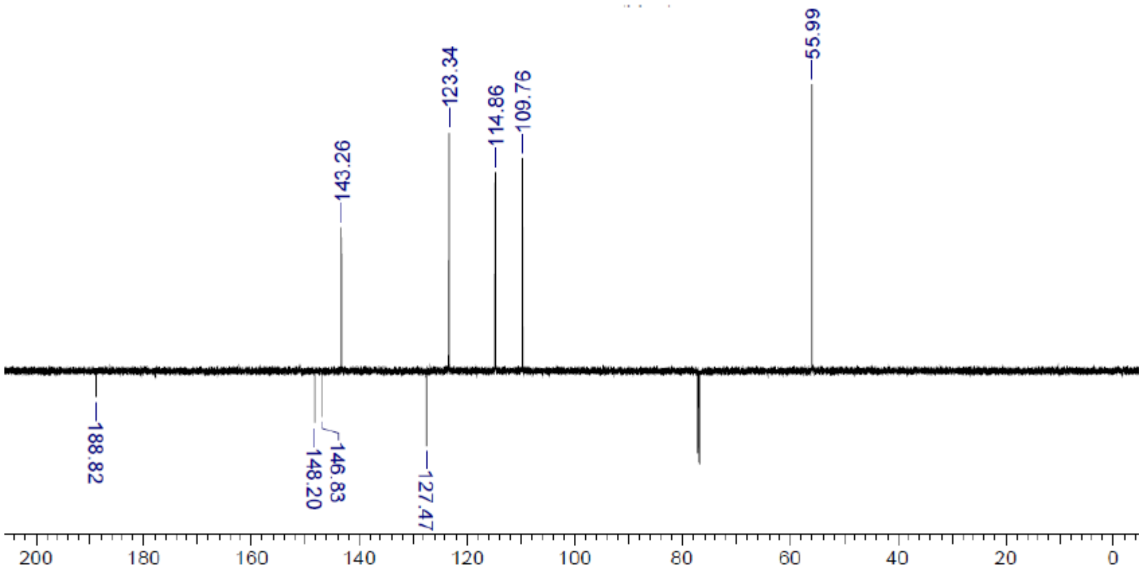

Figure S18. DEPT-Q ^{13}C NMR spectrum (125 MHz, $CDCl_3$) of bis-chalcone (**4**).

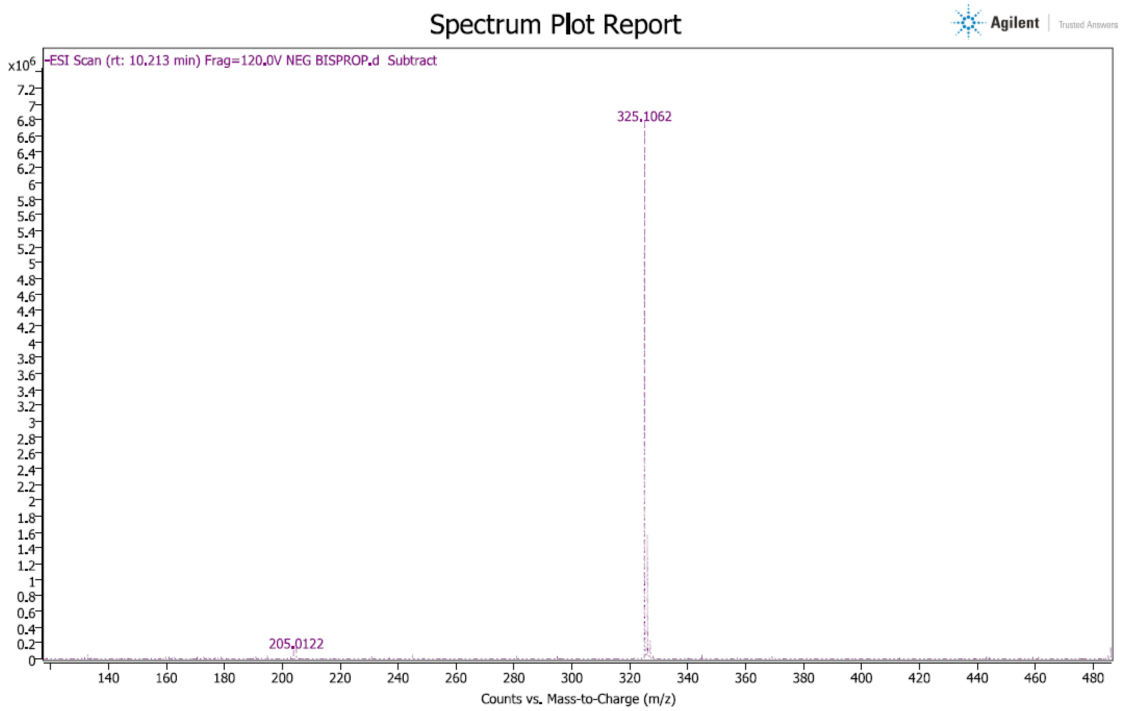

Figure S19. HRMS (Q-TOF) of bis-chalcone (**4**).

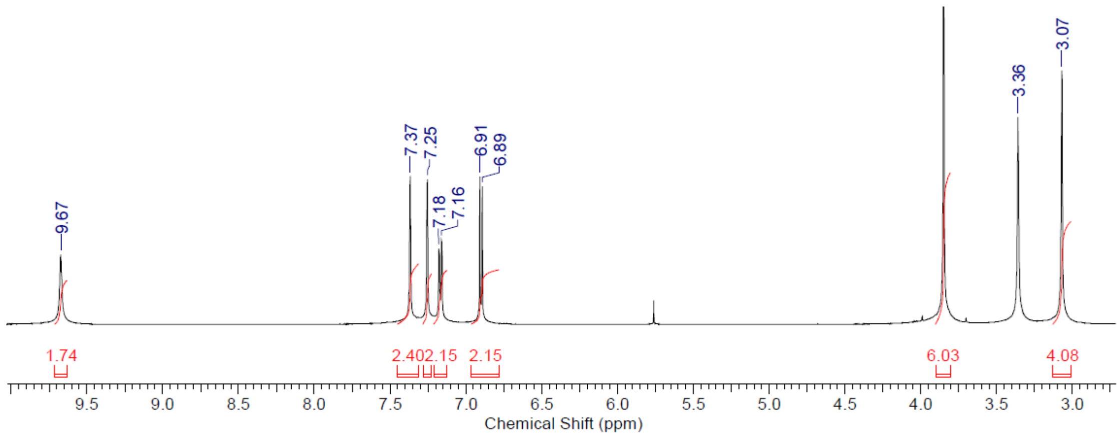

Figure S20. ^{1}H NMR spectrum (500 MHz, DMSO-d_6) of bis-chalcone (**6**).

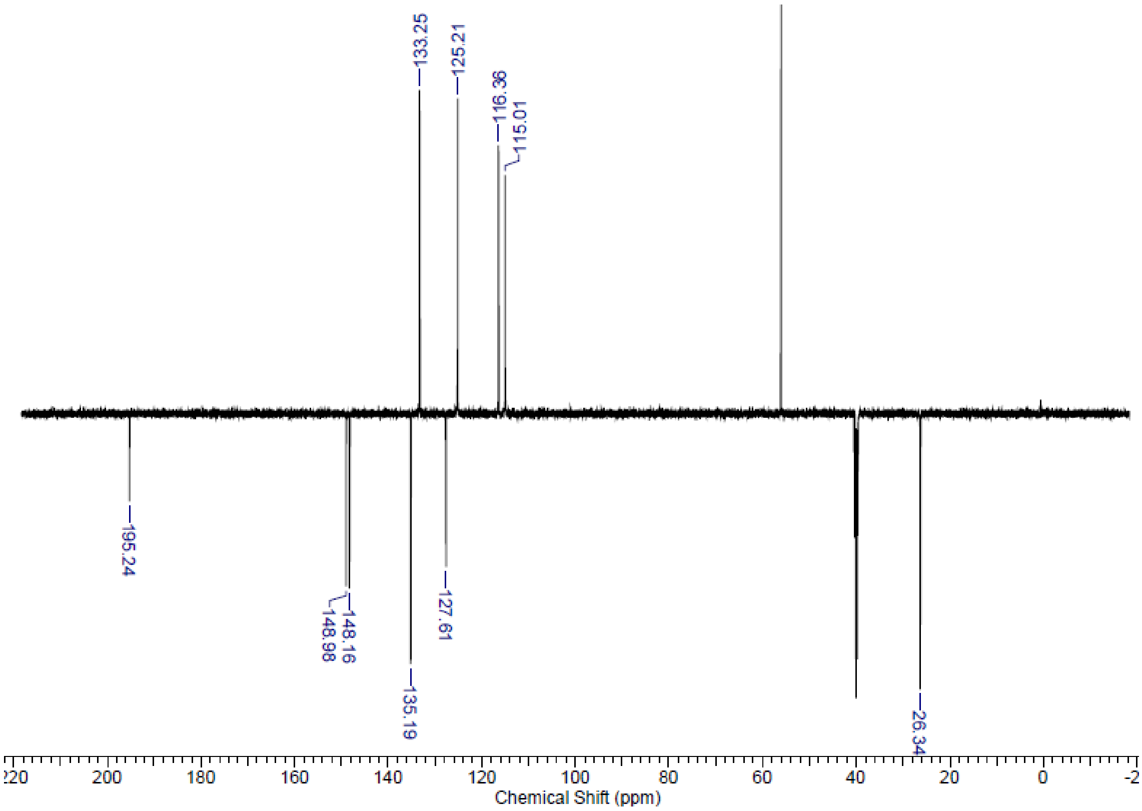

Figure S21. DEPT-Q ^{13}C NMR spectrum (125 MHz, DMSO-d_6) of bis-chalcone (**6**).

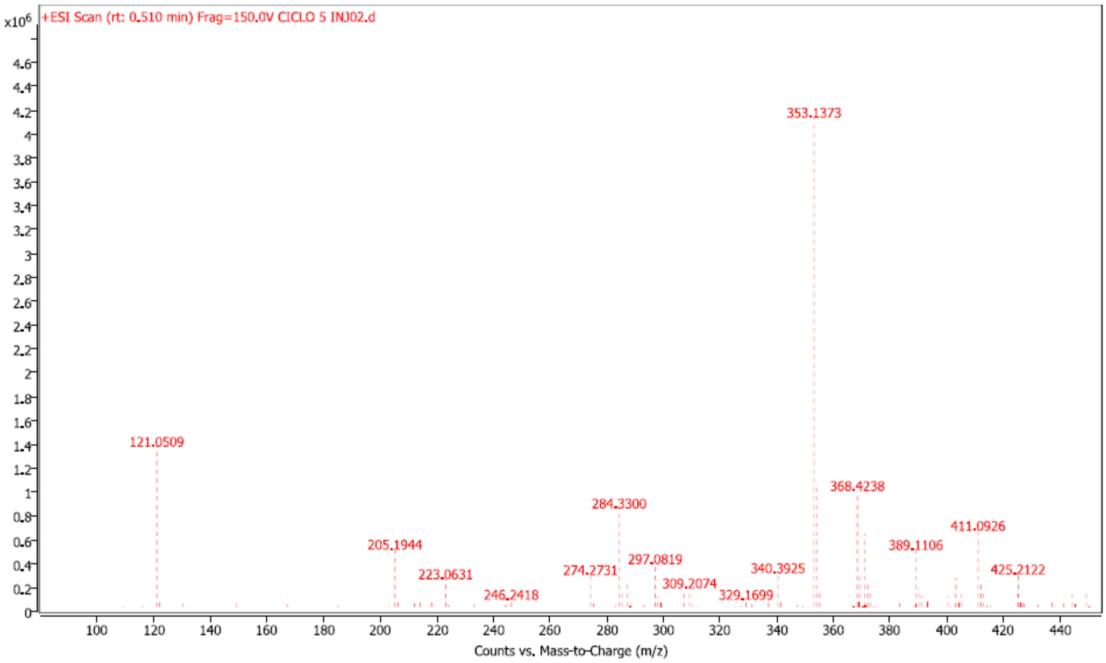

Figure S22. HRMS (Q-TOF) of bis-chalcone (**6**).

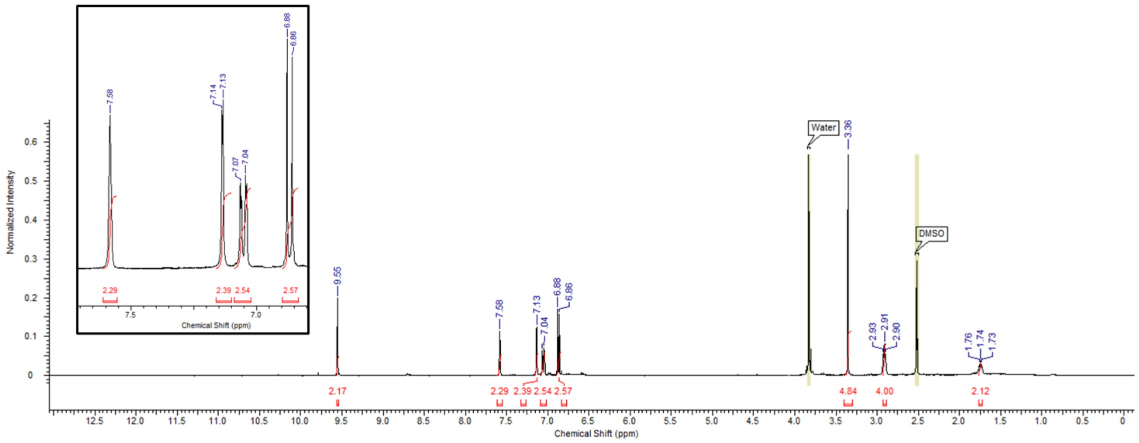

Figure S23. ^{1}H NMR spectrum (500 MHz, DMSO-d_6) of bis-chalcone (**7**).

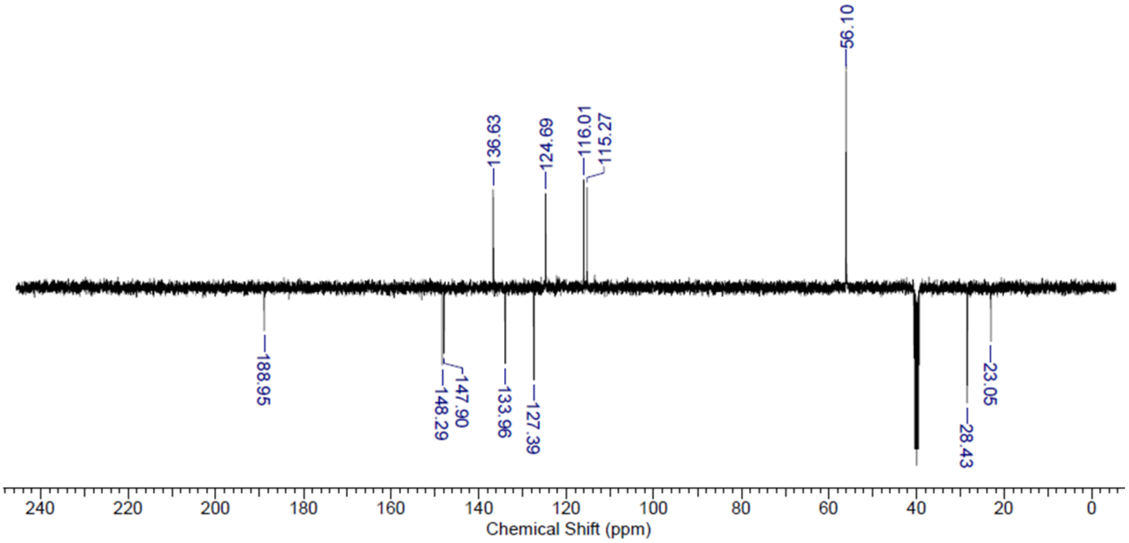

Figure S24. DEPT-Q ^{13}C NMR spectrum (125 MHz, DMSO-d_6) of bis-chalcone (**7**).

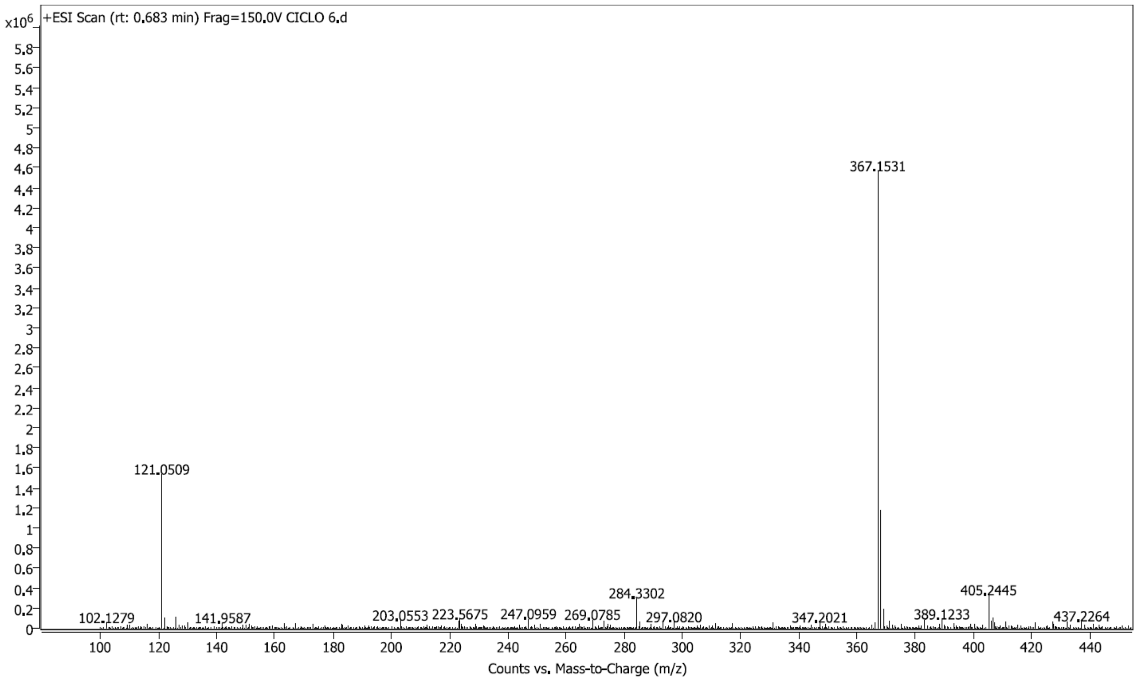

Figure S25. HRMS (Q-TOF) of bis-chalcone (**7**).

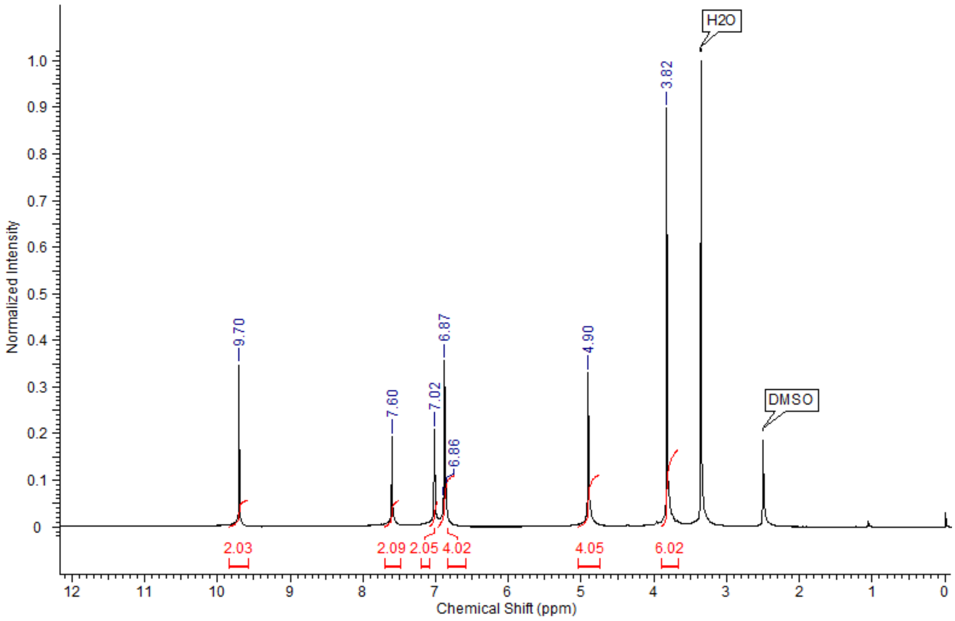

Figure S26. ^{1}H NMR spectrum (500 MHz, DMSO-d_6) of bis-chalcone (**8**).

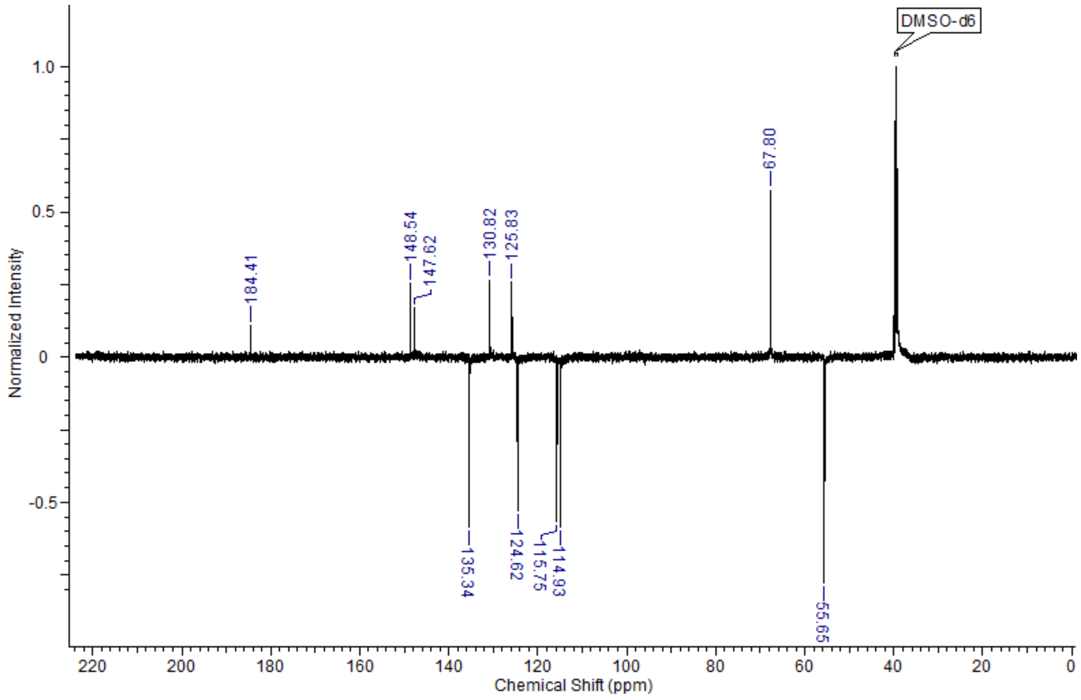

Figure S27. DEPT-Q ^{13}C NMR spectrum (125 MHz, DMSO-d_6) of bis-chalcone (**8**).

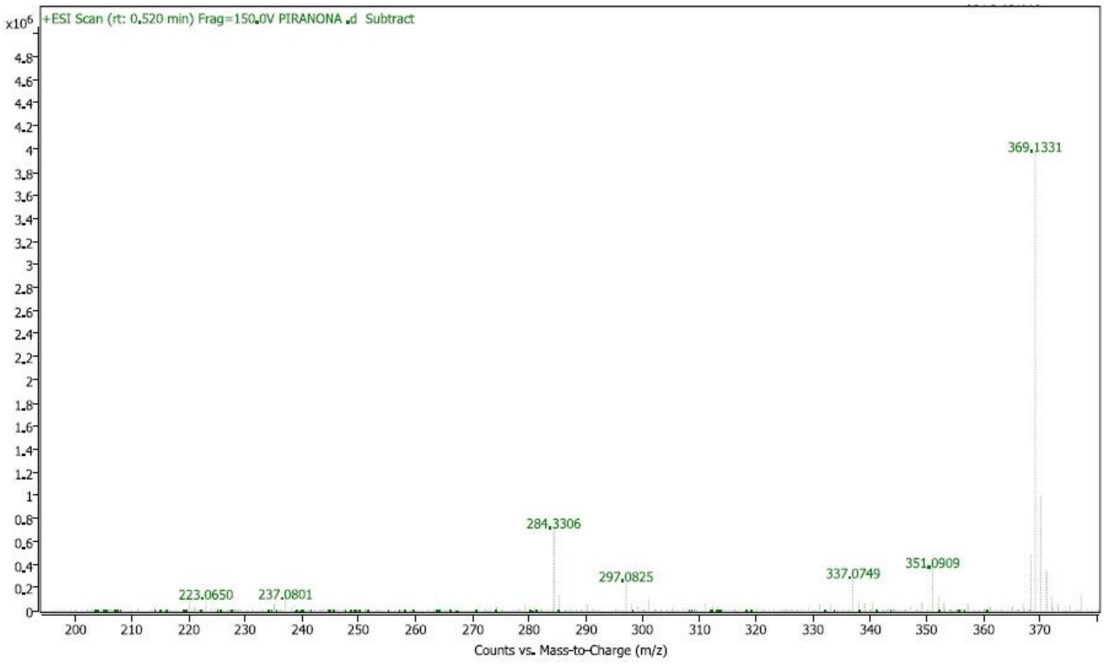

Figure S28. HRMS (Q-TOF) of bis-chalcone (**8**).

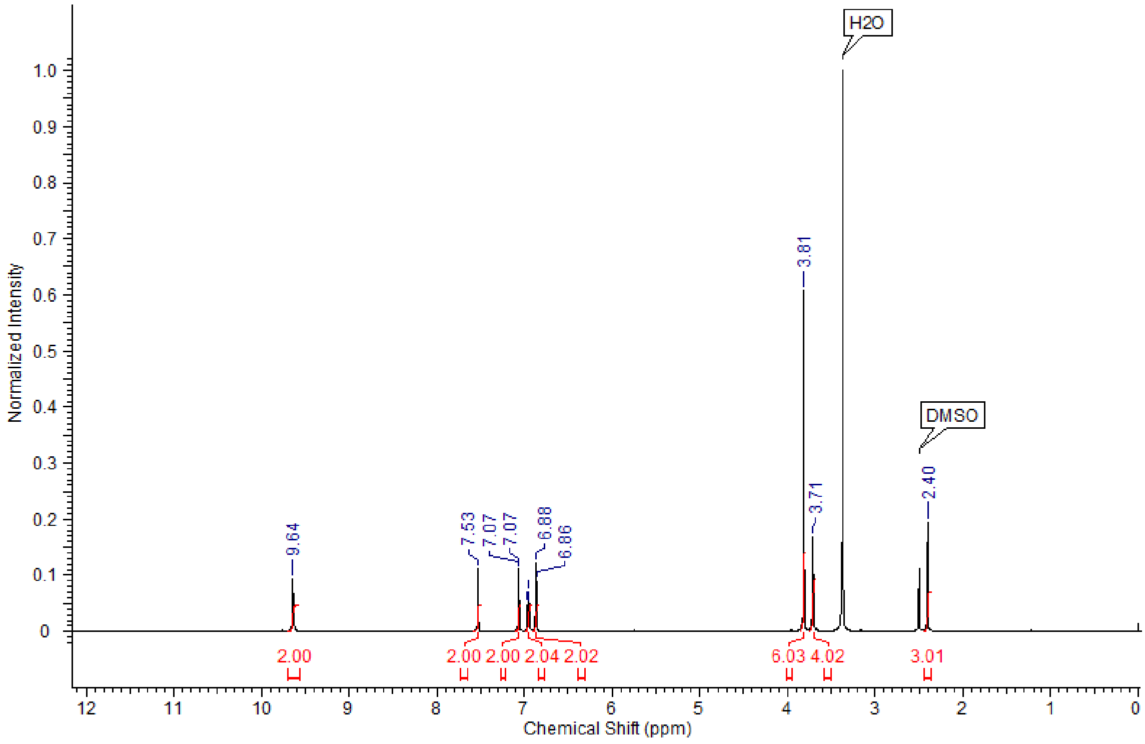

Figure S29. ^{1}H NMR spectrum (500 MHz, DMSO-d_6) of bis-chalcone (**9**).

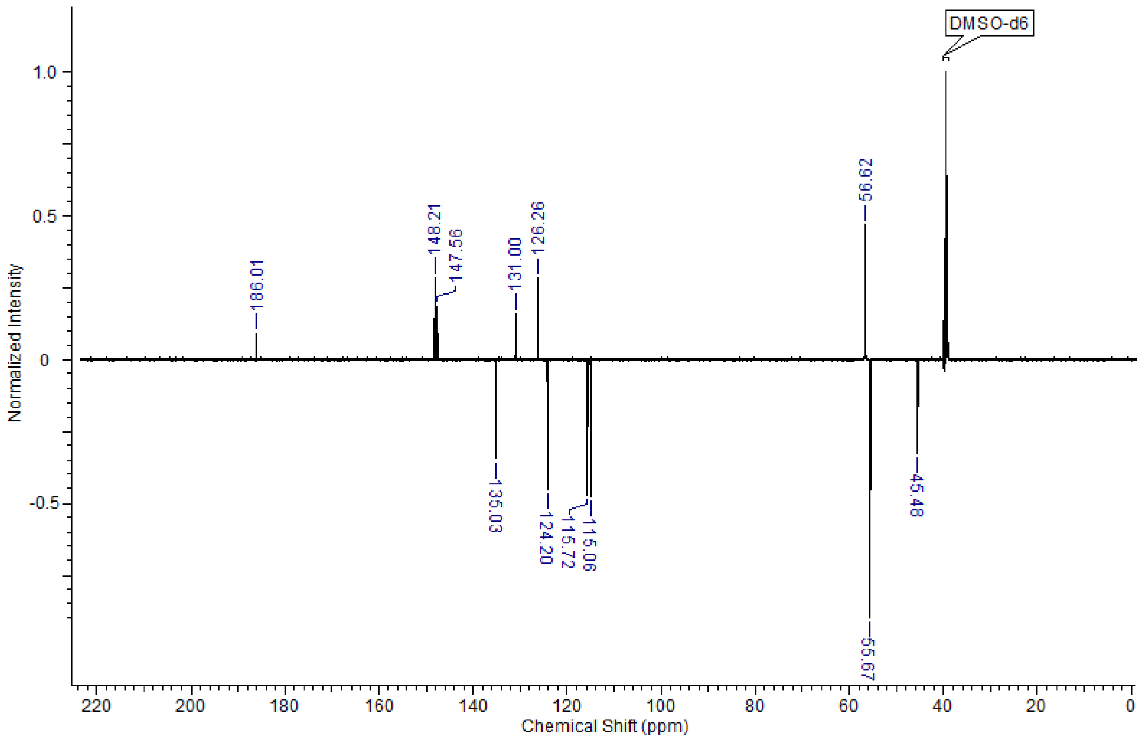

Figure S30. DEPT-Q ^{13}C NMR spectrum (125 MHz, DMSO-d_6) of bis-chalcone (**9**).

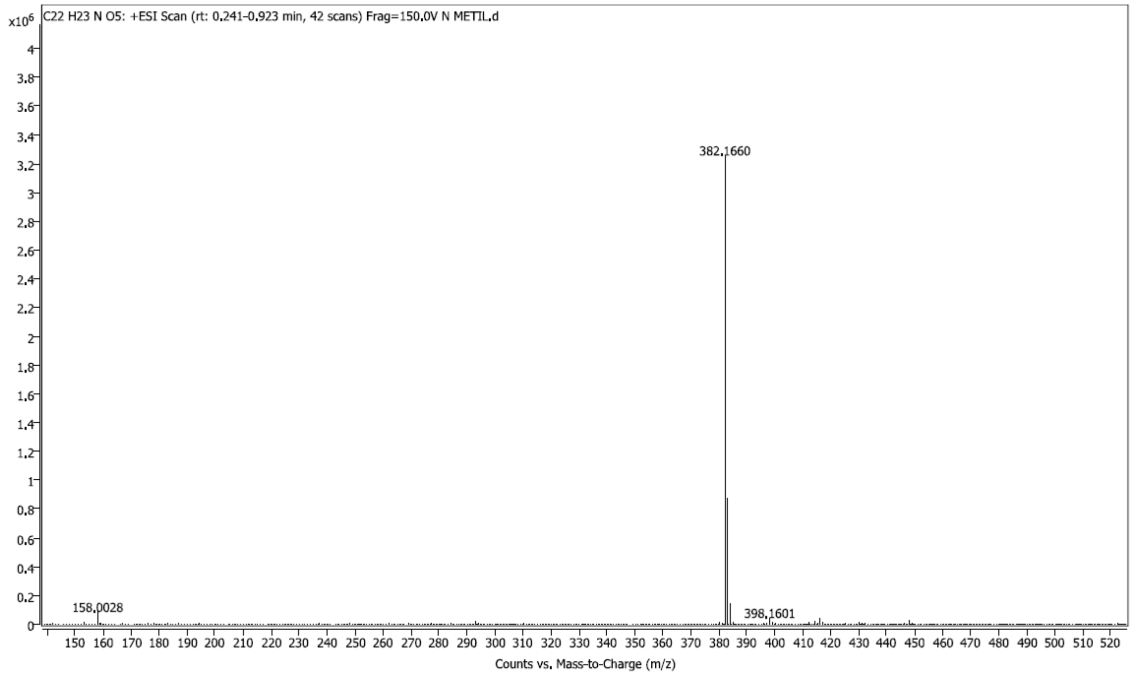

Figure S31. HRMS (Q-TOF) of bis-chalcone (**9**).

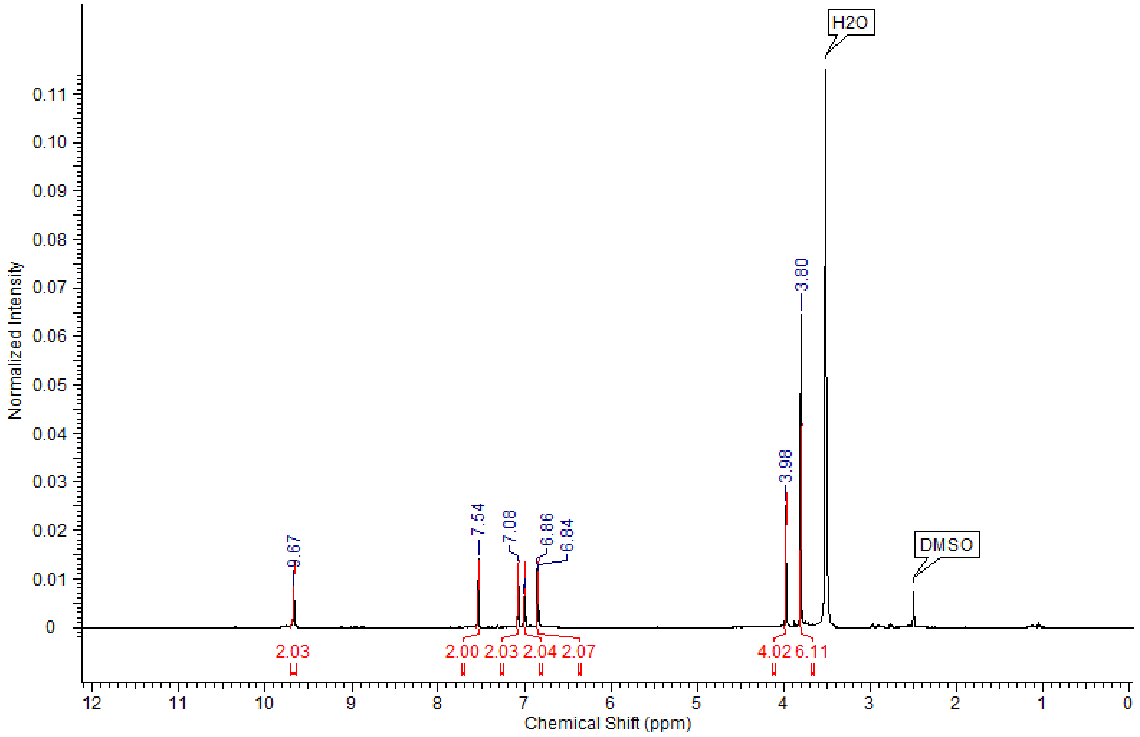

Figure S32. ^{1}H NMR spectrum (500 MHz, DMSO-d_6) of bis-chalcone (**10**).

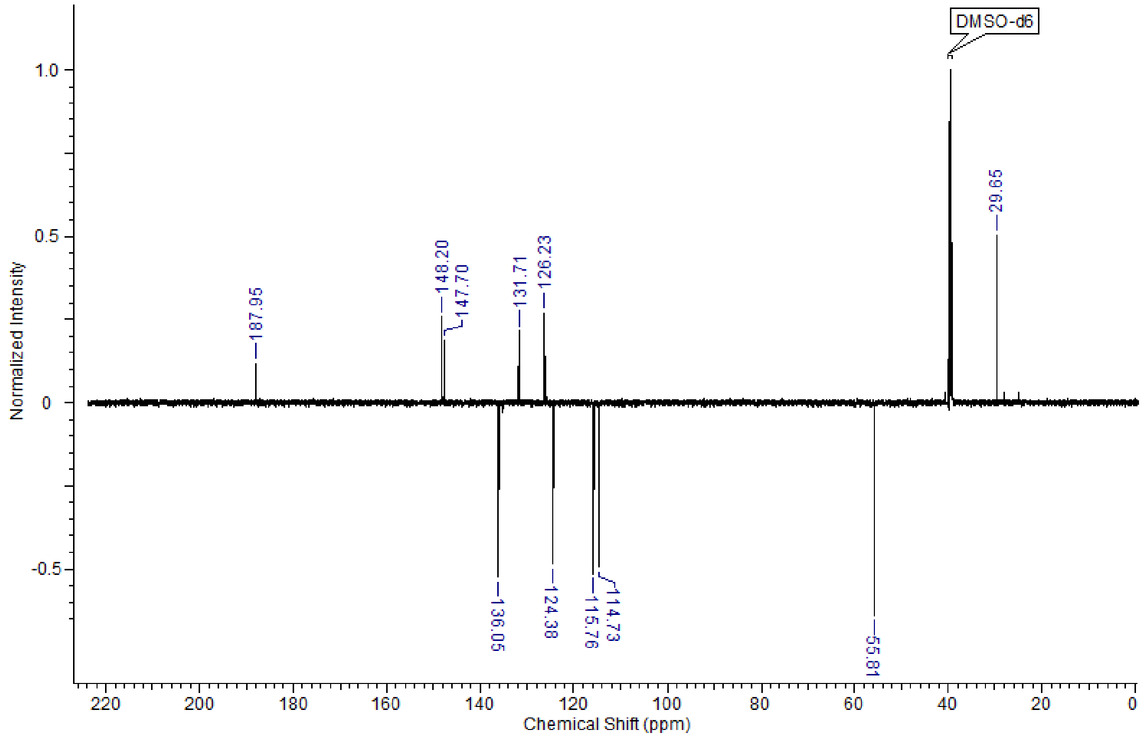

Figure S33. DEPT-Q ^{13}C NMR spectrum (125 MHz, DMSO-d_6) of bis-chalcone (**10**).

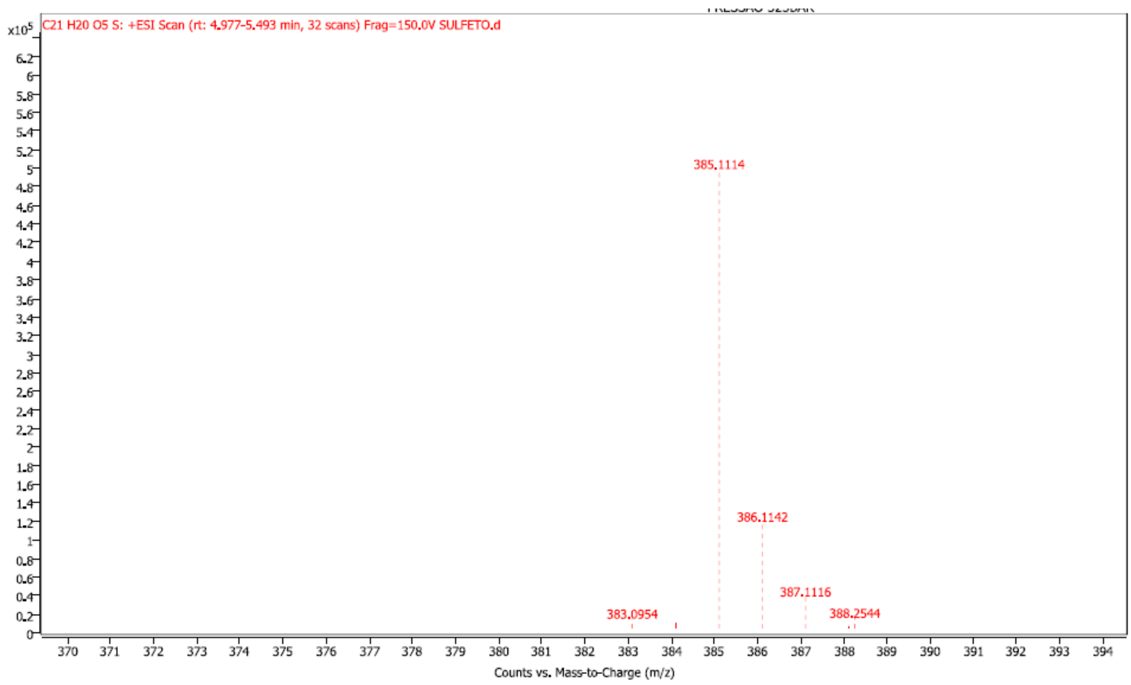

Figure S34. HRMS (Q-TOF) of bis-chalcone (**10**).

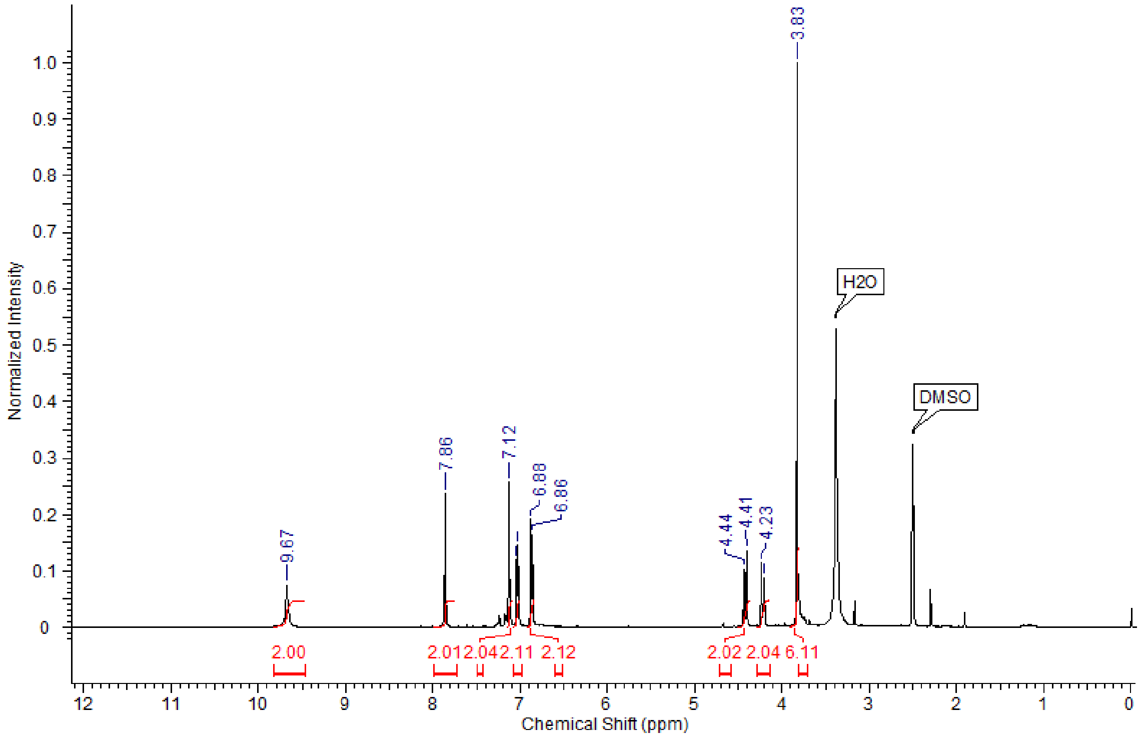

Figure S35. ^{1}H NMR spectrum (500 MHz, DMSO-d_6) of bis-chalcone (**11**).

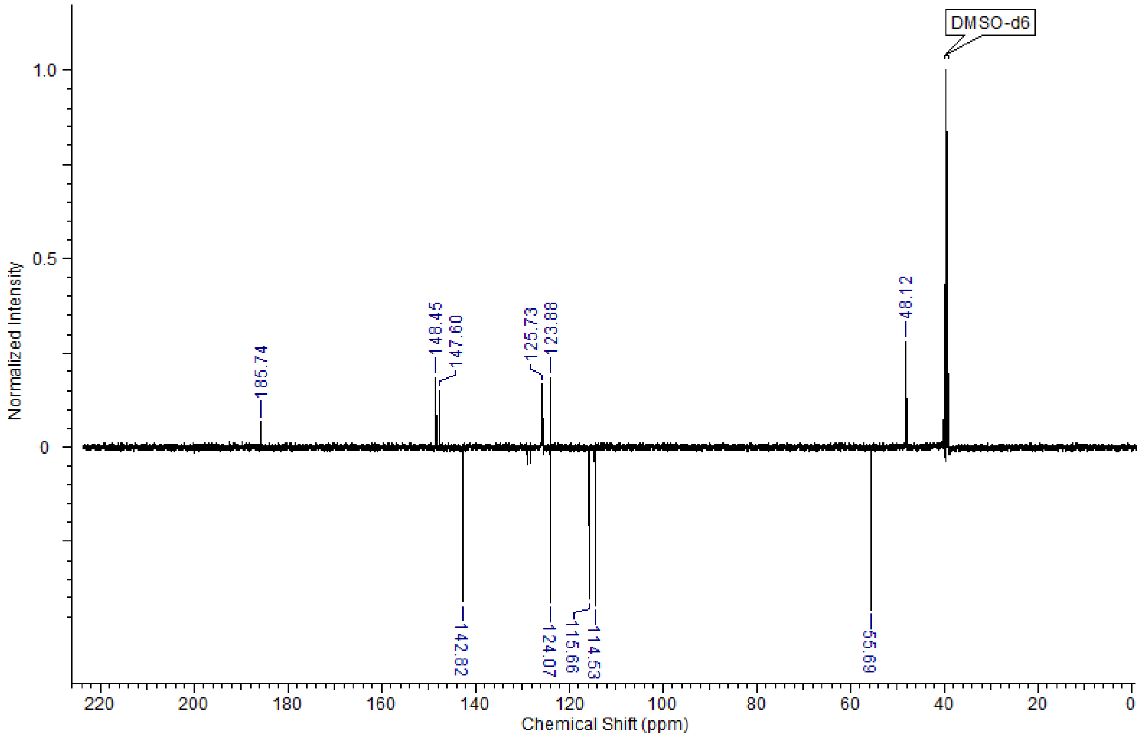

Figure S36. DEPT-Q ^{13}C NMR spectrum (125 MHz, DMSO-d_6) of bis-chalcone (**11**).

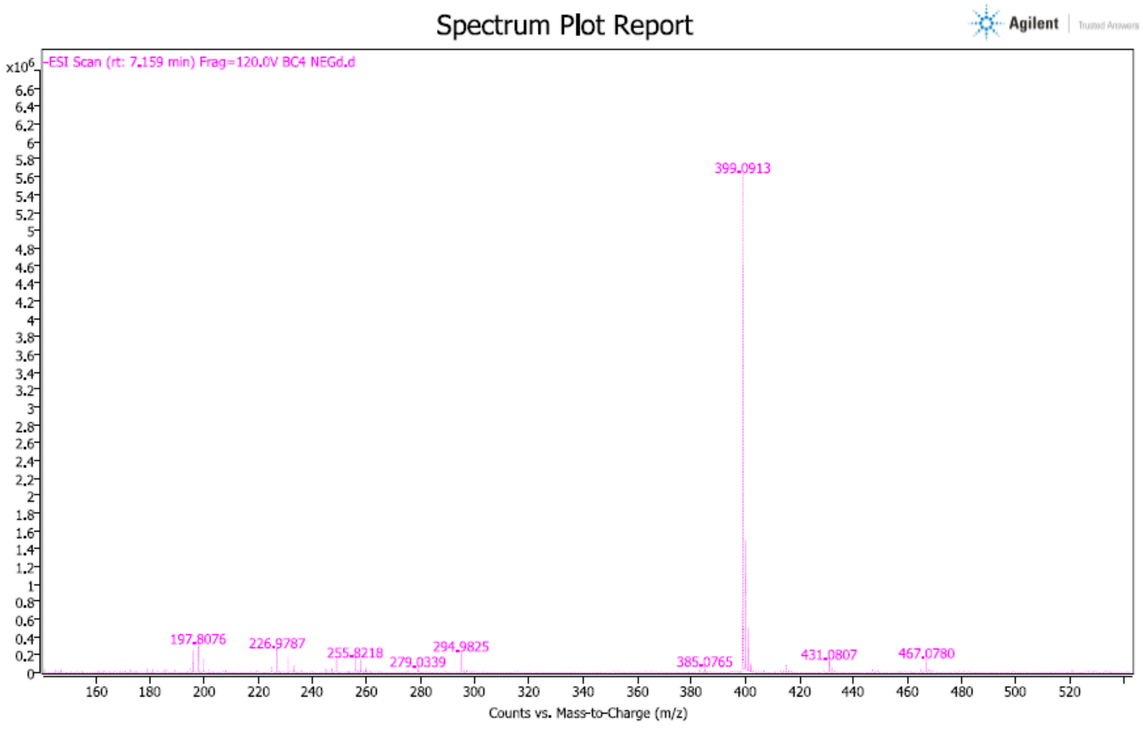

Figure S37. HRMS (Q-TOF) of bis-chalcone (**11**).

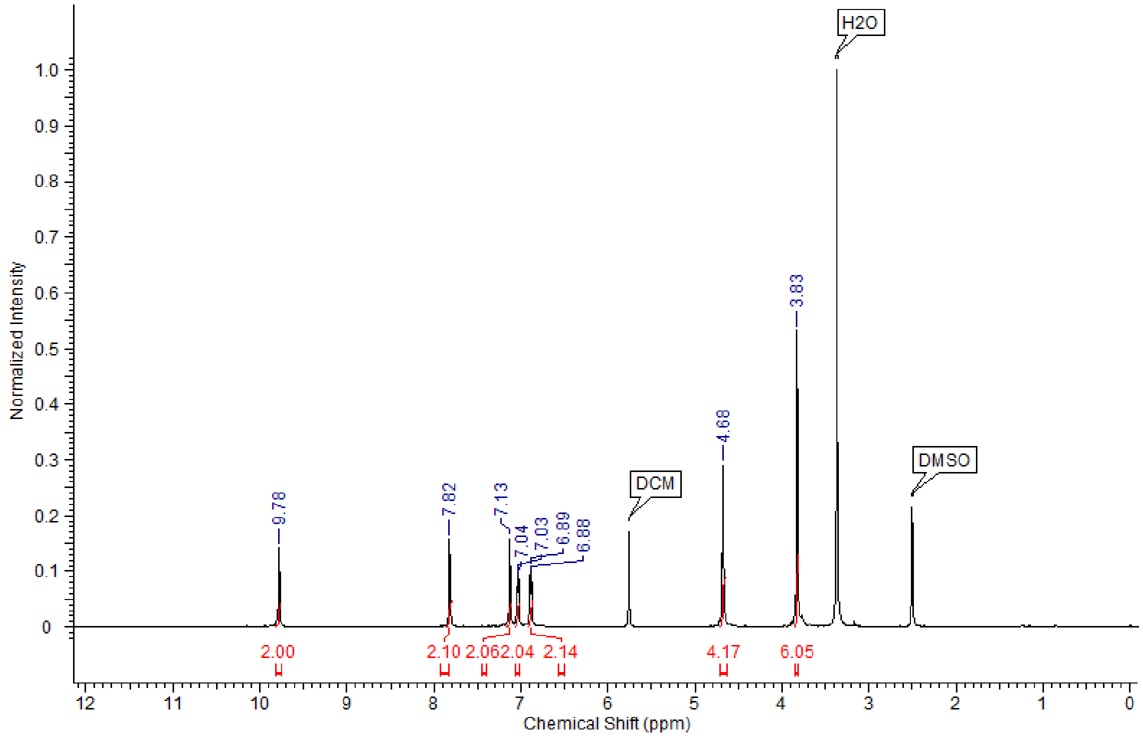

Figure S38. ^{1}H NMR spectrum (500 MHz, DMSO-d_6) of bis-chalcone (**12**).

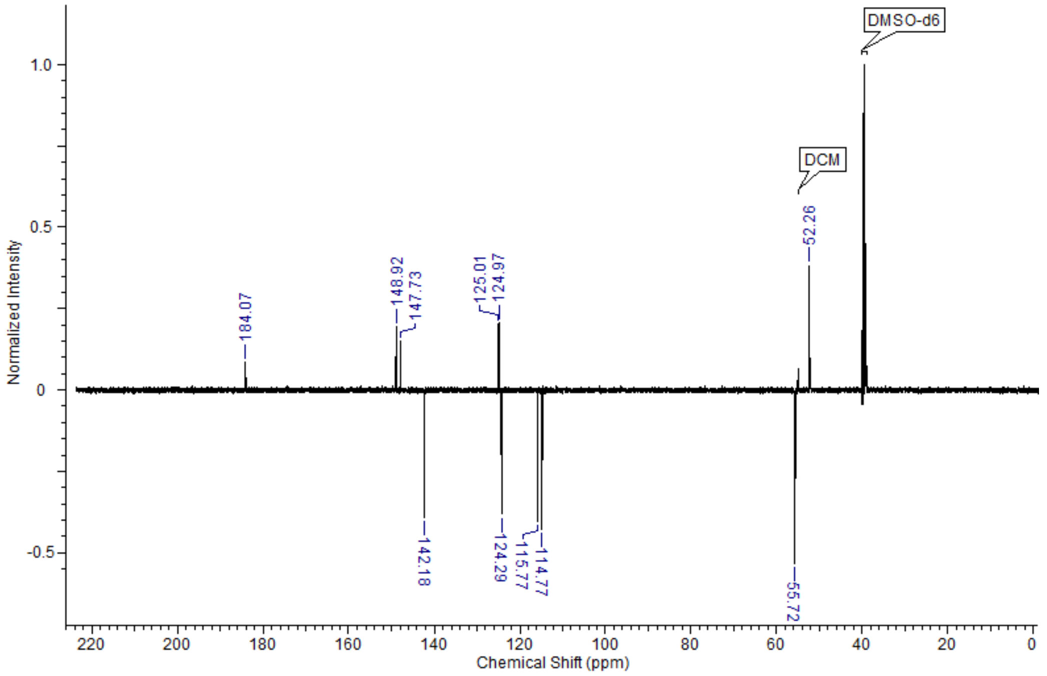

Figure S39. DEPT-Q ^{13}C NMR spectrum (125 MHz, DMSO-d_6) of bis-chalcone (**12**).

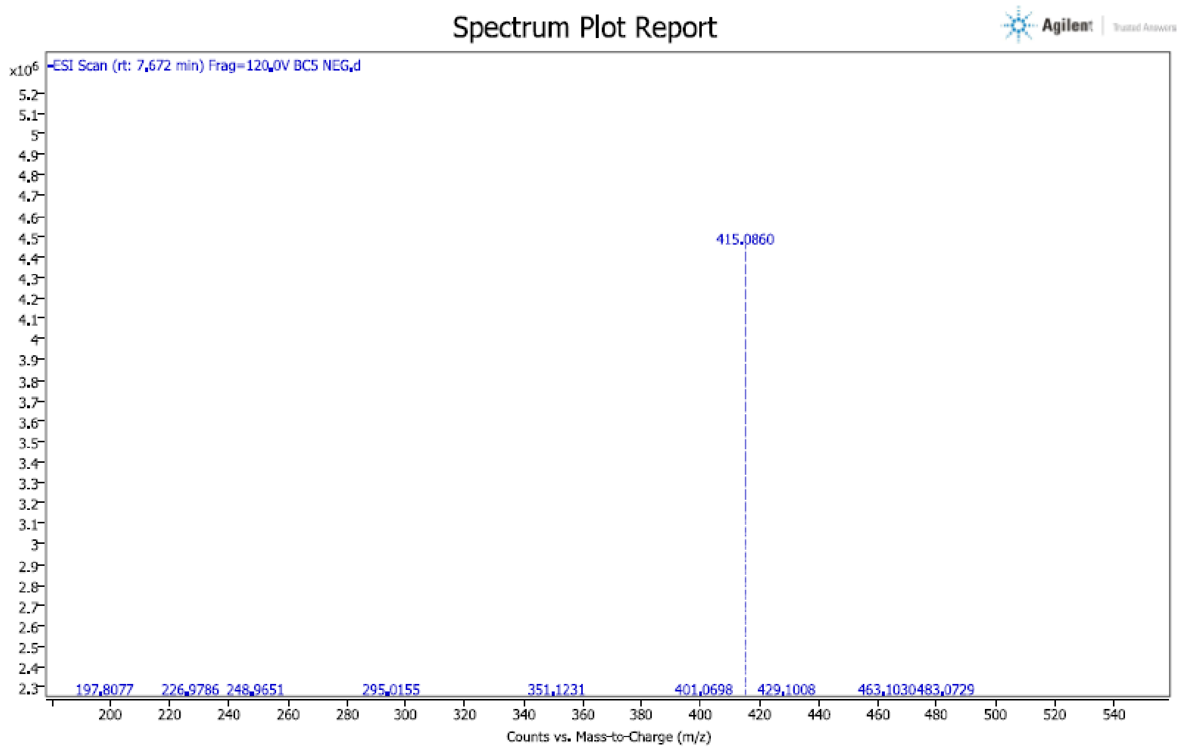

Figure S40. HRMS (Q-TOF) of bis-chalcone (**12**).
